# The Photorespiratory *BOU* Gene Mutation Alters Sulfur Assimilation and Its Crosstalk With Carbon and Nitrogen Metabolism in *Arabidopsis thaliana*

**DOI:** 10.3389/fpls.2018.01709

**Published:** 2018-11-27

**Authors:** Sladjana Samuilov, Dominik Brilhaus, Nadine Rademacher, Samantha Flachbart, Leila Arab, Saleh Alfarraj, Franziska Kuhnert, Stanislav Kopriva, Andreas P. M. Weber, Tabea Mettler-Altmann, Heinz Rennenberg

**Affiliations:** ^1^Chair of Tree Physiology, Institute of Forest Sciences, Faculty of Environment and Natural Resources, University of Freiburg, Freiburg im Breisgau, Germany; ^2^Institute of Plant Biochemistry, Cluster of Excellence on Plant Sciences, Heinrich Heine University, Düsseldorf, Germany; ^3^College of Sciences, King Saud University, Riyadh, Saudi Arabia; ^4^Botanical Institute, Cluster of Excellence on Plant Sciences, University of Cologne, Cologne, Germany

**Keywords:** cysteine synthesis, glutathione, glycine, glycolysis, nitrogen assimilation, photorespiration, serine production, TCA cycle

## Abstract

This study was aimed at elucidating the significance of photorespiratory serine (Ser) production for cysteine (Cys) biosynthesis. For this purpose, sulfur (S) metabolism and its crosstalk with nitrogen (N) and carbon (C) metabolism were analyzed in wildtype *Arabidopsis* and its photorespiratory *bou-2* mutant with impaired glycine decarboxylase (GDC) activity. Foliar glycine and Ser contents were enhanced in the mutant at day and night. The high Ser levels in the mutant cannot be explained by transcript abundances of genes of the photorespiratory pathway or two alternative pathways of Ser biosynthesis. Despite enhanced foliar Ser, reduced GDC activity mediated a decline in sulfur flux into major sulfur pools in the mutant, as a result of deregulation of genes of sulfur reduction and assimilation. Still, foliar Cys and glutathione contents in the mutant were enhanced. The use of Cys for methionine and glucosinolates synthesis was reduced in the mutant. Reduced GDC activity in the mutant downregulated Calvin Cycle and nitrogen assimilation genes, upregulated key enzymes of glycolysis and the tricarboxylic acid (TCA) pathway and modified accumulation of sugars and TCA intermediates. Thus, photorespiratory Ser production can be replaced by other metabolic Ser sources, but this replacement deregulates the cross-talk between S, N, and C metabolism.

## Introduction

Sulfate assimilation constitutes the source of reduced sulfur for the synthesis of cysteine and numerous primary and secondary metabolites (Koprivova and Kopriva, 2014). Therefore, sulfate acquired by the roots is transported in the xylem sap to the leaves and allocated to the chloroplasts for reduction and assimilation (Takahashi et al., 2011). Cysteine (Cys) is the terminal product of sulfur assimilation. It is synthesized in the chloroplast, the mitochondria and the cytosol (Takahashi et al., 2011) from sulfide in a two-step reaction: In the first step, serine acetyltransferase (SAT) catalyses the synthesis of O-acetylserine (OAS) from serine (Ser) and acetyl CoA, while in the second step O-acetyl-serine (thiol) lyase (OAS-TL) mediates the synthesis of Cys by incorporation of sulfide into OAS (Takahashi et al., 2011; Rennenberg and Herschbach, 2014). SAT and OAS-TL form the Cys synthetase complex (CSC), which plays a regulatory role in Cys synthesis by protein–protein interactions of its constituent enzymes (Wirtz et al., 2012).

Beside its requirement for protein synthesis, Cys is an important precursor of all thiol containing organic sulfur compounds, including glutathione (GSH), methionine (Met), and glucosinolates (GLS). With its thiol group, GSH is involved in numerous redox reactions (Foyer and Noctor, 2000), but also constitutes the storage and long-distance transport form of reduced sulfur (Herschbach et al., 2012). In addition, it plays an important role in scavenging reactive oxygen species in the ascorbate-glutathione cycle (Noctor et al., 2012), in the detoxification of xenobiotics by conjugation *via* GSH-S transferases (Gullner et al., 2001) and for the sequestration of heavy metals as metabolic precursor of phytochelatins that function as heavy metal chelators (Gupta et al., 2010; He et al., 2015). Met is another important sink of Cys from its cytosolic, plastidic and/or mitochondrial pool. It serves as a building block for protein, *S*-adenosyl methionine (SAM) and S-methylmethionine (SMM) biosynthesis (Amir et al., 2002). Glucosinolates are secondary sulfur-rich metabolites with a defense function against herbivores and pathogens (Brader et al., 2006), characteristic for *Brassicaceae* (Huseby et al., 2013).

Cys synthesis requires a cross talk of sulfur assimilation with nitrogen and carbon metabolism (Kopriva and Rennenberg, 2004). The carbon skeleton and the nitrogen in Cys both originate from Ser. 3-phosphoglycerate (3-PGA), an intermediate of glycolysis and 2-phospoglycolate, a product of photorespiration, are sources of the carbon skeleton in Ser, while nitrogen is a product of nitrate reduction and assimilation (Kopriva and Rennenberg, 2004). Since nitrogen assimilation is catalyzed by the GS-GOGAT pathway that requires 2-oxoglutarate for glutamate synthesis, reactions of the tricarboxylic acid (TCA) cycle in mitochondria are also involved in providing Ser for Cys synthesis (Kopriva and Rennenberg, 2004). Ser biosynthesis takes place in different cellular compartments, i.e., in plastids, the mitochondria and the cytosol. Its synthesis is achieved by three distinct alternative routes, a photorespiratory and two non-photorespiratory pathways, i.e., the phosphorylated pathway and the glycerate pathway (Ros et al., 2013). The mitochondrial photorespiratory pathway of Ser biosynthesis is currently considered the dominant biosynthetic route of Ser synthesis in photosynthetic cells, while the phosphorylated pathway is supposed to predominantly proceed in plastids of non-photosynthetic tissues or in photosynthetic tissue during night (Benstein et al., 2013). An *Arabidopsis* transgenic line, deficient in phosphoglycerate dehydrogenase (PGDH) activity showed reduced growth that can be restored by external provision of Ser. In addition, when this mutant was cultivated at high CO_2_ conditions, which suppresses photorespiration, reduced growth and development of new leaves were observed (Benstein et al., 2013). This finding supports the idea that photorespiratory Ser production is the dominant route of Ser biosynthesis in leaves. The *Arabidopsis* mutant *shm1-1*, defective in mitochondrial Ser hydroxymethyltransferase (*SHMT*), can survive only in elevated CO_2_ (Voll et al., 2006). The *SHM2* isozyme of the *SHMT* gene is expressed in vascular tissue of leaves and, in contrast to the *shmt1-1* mutant, the *shm2-2* mutant, deficient in the *SHM2* gene, does not display a visible phenotype in ambient air (Engel et al., 2011). However, the double mutant (*shm1* × *shm2*) cannot survive without Suc supplementation even at elevated CO_2_, indicating that the presence of at least one isozyme, either *SHMT1* or *SHMT2*, is essential for non-photorespiratory carbon metabolism (Engel et al., 2011).

For studying the significance of the photorespiratory pathway of Ser biosynthesis in Cys production, the *Arabidopsis* “*a bout de soufflé*” (*bou-2*) mutant, with reduced activity of the mitochondrial glycine decarboxylase (GDC) multi-enzyme due to knock-down of mitochondrial BOU transporter protein, seems to be a suitable subject. The *BOU* gene is strongly co-expressed with the genes *GDP2*, *GDP1*, *GDT*, and *HGDH3* encoding subunits of the *GDC* multi-enzyme complex, and *SHM1*, encoding mitochondrial *SHM1* (Eisenhut et al., 2013). In experiments with the *Arabidopsis bou-2* mutant, the level of *GDP* protein was decreased, which led to a strongly reduced *GDC* activity (Eisenhut et al., 2013). Although *bou-2* plants are not lethal, they show severe growth defects at ambient CO_2_, such as slow shoot apical meristem cell cycle activity and cell division (Eisenhut et al., 2013). After 2 days of growth at elevated CO_2_, cell division was resumed in *bou-2* mutants, indicating the requirement of suppression of photorespiration by elevated CO_2_ for growth and development of the mutant (Eisenhut et al., 2013).

The aim of the present study was (1) to elucidate the significance of chloroplastic and mitochondrial pathways of Ser production for Cys biosynthesis, and its use for protein, GSH and GLS production and (2) to identify genes involved in the crosstalk between sulfur and nitrogen metabolism. Experiments were conducted with *Arabidopsis* wild type (WT) plants as control in comparison to *bou-2* plants with impaired photorespiration. It is hypothesized that despite different cellular localization of Cys biosynthesis (chloroplast, mitochondria, and cytosol), photorespiratory (mitochondrial) Ser is the dominant precursor of Cys synthesis in photosynthetic tissue during light (hypothesis 1). Further it is hypothesized that Ser biosynthesis in chloroplasts/plastids takes over Ser production for Cys biosynthesis in photosynthetic tissue in the dark (hypothesis 2). As third hypothesis it is assumed that these differences in Ser supply for Cys biosynthesis between day and night are regulated by gene expression. To address these hypotheses, S-flux experiments combined with metabolome and RNAseq analyses were performed.

## Materials and Methods

### Plant Material and Growth Conditions

*Arabidopsis thaliana* Heynh. Col–0 and its mutant *bou-2* (Li et al., 2003; Rosso et al., 2003), available from the Nottingham *Arabidopsis* Stock Centre as N370142 and harboring a T-DNA insertion in the second exon of the *BOU* gene sequence, were used in this study. Bioinformatic analysis confirmed that the *bou-2* mutant line contains a single T-DNA insertion exclusively for the locus At5g46800 encoding *BOU* transporter protein (see Supplementary Method S1). For sterilization, *Arabidopsis* seeds were washed twice with 70% (v/v) ethanol, containing 1% (v/v) Triton X-100, twice with 100% ethanol and dried. Seeds were grown on 0.5 Murashige and Skoog (MS) medium (Murashige and Skoog, 1962) with 0.8% (w/v) agar at pH 5.8. After cold stratification at 4°C to synchronize germination, seedlings were grown at 60% relative humidity, 100–150 μmol photons m^-2^ s^-1^, 12 h light / 12 h dark, 22°C / 18°C in a 3,000 ppm CO_2_-enriched atmosphere. Seedlings with two developed leaves were transferred to soil and cultivated 4 more weeks under the conditions indicated above. Experiments were conducted with plants of similar developmental stage.

### Experimental Design

*Arabidopsis wildtype* (WT) *and bou-2 mutant plants* were transferred to ambient CO_2_ (400 ppm) 24 h prior to harvest. Two sets of plants were harvested (1) ca. 10 min after light onset and (2) 3 h before light onset. The whole rosette of the first set of plants was used for quantification of metabolites and for RNAseq analyses. From the other set of plants fully mature leaves (number 5 to 7 counted by the order of emergence from the rosette) were cut. Half of these leaves were exposed to [^35^S]-sulfate for analysis of total sulfate uptake, the flux into the pools of sulfate, proteins, GSH and GLS, as well as GSH and GLS contents. The other half of the leaves was treated in the same way with non-radioactive Hoagland solution and was used for the determination of sulfate and protein contents. Leaf samples were frozen in liquid nitrogen and stored at -80°C until further use.

### [^35^S]-Sulfate Feeding Experiment and Analysis of [^35^S]-Uptake

Before conducting [^35^S]-sulfate feeding experiments, a preliminary test with tree time points (2, 4, and 6 h) of incubation with labeled SO_4_^2-^ was performed in order to confirm linearity of uptake (data not shown). From these preliminary tests, a 4 h incubation time was found to be well in the linear phase of sulfate uptake. For [^35^S]-sulfate feeding experiments, leaves were transferred into tubes (Sarstedt AG & Co., Nümbrecht, Germany) with 10 mL of Hoagland solution (5 mM KNO_3_, 5 mM Ca(NO_3_)_2_, 2.5 mM KH_2_PO_4_, 2.5 mM KOH, 25 μM MnCl_2_, 2.5 μM ZnSO_4_, 1.25 μM CuSO_4_, 0.075 mM H_3_BO_3_, 2.5 μM Na_2_MoO_4_, 1.25 μM Co(NO_3_)_2_, and 0.0675 mM Fe-EDTA) adjusted to 0.2 mM SO_4_^2-^ for 2 h of pre-incubation. Thereafter, leaves were placed for 4 h in new Hoagland solution with [^35^S]-SO_4_^2-^ (Hartman Analytic, Braunschweig, Germany) at 50 μCi (1.85 × 10^-6^ Bq) for day and 100 μCi (3.7 × 10^-7^ Bq) for night. At the beginning of the incubation, leaves were placed in a desiccator and infiltrated with a vacuum pump to exchange air from the apoplastic space with radioactive Hoagland solution. At the end, sulfate uptake was stopped by washing the leaves with 10 mL non-radioactive incubation solution. For night experiments, pre-incubation and incubation were performed in the dark; night harvests were carried out under green light (GreenHornet LED, 3.5W; Dutch-Headshop Services, Beverwijk, Netherlands). Leaf tissue was homogenized with a micro pestle and mortar, extracted with 0.1 M HCl at a ratio of 1:10 (w/v). Aliquots of 10 μL extract were mixed with 2 mL of Rotiszint^®^ eco plus (Carl Roth, Karlsruhe, Germany) scintillation cocktail and radioactivity was determined with a scintillation counter (LS 6500 Multi-Purpose Scintillation Counter, Beckman Coulter, Brea, CA, United States). From the data obtained and the specific radioactivity of the incubation solution, sulfate uptake was calculated.

### [^35^S]-Metabolite Analyses and Determination of Flux Through the Sulfate Assimilation Pathway

The flux through the sulfate assimilation pathway was calculated as incorporation of ^35^[S]-sulfate into GSH, proteins and GLS, as previously described by Scheerer et al. (2010) and Mugford et al. (2011).

### Protein Contents and [^35^S] Flux Into Proteins

For protein analysis, 30 μL of a fresh prepared solution containing dithiothreitol (DTT, 500 mM), phenylmethylsulfonylchloride (PMSF, 100 mM) and 10% Triton x-100 were added into 2 mL micro tubes (Sarstedt, Nümbrecht, Germany) containing a mixture of 50 mg of pre-washed polyvinylpolypyrrolidone (PVP 6755, Merck, Darmstadt, Germany) and 500 μL buffer solution (50 mM Tris–HCl, pH 8.0, 1 mM EDTA, 15% glycerol (v/v)) previously incubated overnight at 4°C. Approximately 30 mg ground leaf material were added, suspended by vortexing and incubated for 30 min at 4°C. After centrifugation at 15,000 × *g* for 10 min at 4°C, 200 μL supernatant were transferred into new tubes and 200 μL trichloroacetic acid (TCA, 10%) were added. Samples were incubated at room temperature for 10 min and centrifuged at 15,000 × *g* and 4°C. The supernatant was discarded. The pellet was dissolved in 500 μL 1 M KOH by shaking for 30 min at 4°C. The protein content was determined as described by Bradford (1976) using dilutions of 1 mg mL^-1^ bovine serum album (BSA) (Merck, Darmstadt, Germany), dissolved in 1 M KOH as a standard. Micro test plates (96 Well, Sarstedt, Nümbrecht, Germany) were filled with 200 μL Bradford Reagent (Merck, Darmstadt, Germany) and aliquots of 10 μL samples and standards were added in triplicates. After 10 min incubation at room temperature in the dark, the optical density was measured at a wavelength of 595 nm using an ELISA reader (Rainbow Thermo Reader, Tecan, Crailsheim, Germany). To determine radioactivity in proteins, aliquots of 50 μL supernatant previously extracted with 0.1 M HCl (see: [^35^S]-sulfate feeding experiment and analysis of [^35^S]-uptake) were transferred into new 1.5 mL micro tubes (Sarstedt, Nümbrecht, Germany). Protein was precipitated with 12.5 μL 100% trichloracetic acid (TCA) as described by Kopriva et al. (1999). After 15 min on ice, 100 μl 100% TCA was added and the precipitate was collected by centrifugation (at 15,000 × *g* for 10 min). The supernatants were discarded and the pellets were washed with 200 μL 100% ethanol. Subsequently, pellets were dissolved in 100 μL 0.1 M NaOH. Radioactivity was determined in 200 μL supernatant mixed with 2 mL Rotiszint^®^ eco plus scintillation cocktail (Carl Roth, Karlsruhe, Germany).

### Thiol Contents and [^35^S] Flux Into Thiols

Total thiol contents were determined as previously described by Schupp and Rennenberg (1988) and modified by Strohm et al. (1995). For this purpose, aliquots of 25 μL supernatant extracted with 0.1 M HCl (see: [^35^S] sulfate feeding experiment and analysis of [^35^S] uptake) were transferred into 1.5 mL micro tubes (Sarstedt, Nümbrecht, Germany) and neutralized by adding an equal volume of 0.1 M NaOH. Reduction of thiols was performed with dithiothreitol (DTT; 1 μl, 100 mM) for 15 min in the dark at 37°C. Aliquots of 11.5 μL 1 M Tris buffer and 35 μl distilled water were added. After 15 min of derivatization with monobromobimane (5 μl, 100 mM) in the dark at 37°C, thiols were stabilized by adding 100 μL acetic acid (9%, v / v). Aliquots of 100 μL derivatized solution were injected into a HPLC system (Dionex UltiMate 3000; Thermo Fisher, Waltham, United States) and thiol derivatives were separated on a Spherisorb^®^ ODS2(C_18_) (250 × 4.6 mm, 5 μm particle size) column (Waters, Milford, CT, United States), using a solution of 0.25% (v / v) acetic acid and 10% (v / v) methanol as buffer A and 0.25% acetic acid and 90% (v / v) methanol as buffer B. Thiols were detected fluorometrically (474 Fluorescence detector; Waters, Milford, CT, United States) with excitation at 390 nm and emission at 480 nm. ^35^[S] incorporation into thiols was detected by a radioactivity detector module (FlowStar LB 513; Berthold Technologies, Bad Wildbad, Germany) connected to the HPLC.

### GLS Analysis and [^35^S] Flux Into GLS

The ^35^[S] flux into GLS and GLS contents were quantified after extraction of leaf tissue with 500 μL 70% (v/v) methanol as described by Brown et al. (2003). Aliquots of 10 μL sinigrin hydrate (Merck, Darmstadt, Germany) were added to leaf extracts as internal standard. After 45 min incubation at 70°C, supernatants were placed on previously prepared sephadex columns (DEAE Sephadex^TM^ A-25, GE Healthcare Bio-Sciences, Uppsala, Sweden) and washed with sterile ddH_2_O. Then 75 μL sulfatase (Sigma-Aldrich, Steinheim, Germany) were added to the columns and left overnight at room temperature. Subsequently, GLS were eluted with 1 mL sterile ddH_2_O. The eluate was collected in 1.5 mL micro tubes with caps (Sarstedt, Nümbrecht, Germany), vortexed and centrifuged 5 min at 15,000 × *g*. For determination of GLS, 50 μL supernatant were injected into a HPLC system (Dionex UltiMate 3000; Thermo Fisher, Dreieich, Germany) and separated on a Spherisorb^®^ ODS2(C18) (250 × 4.6 mm, 5 μm particle size) column, using distilled water (solvent A) and 100 % acetonitrile (solvent B) for elution. Quantification was based on UV absorption at 229 nm and the response factor of the internal standard. To determine ^35^[S] incorporation into GLS, 300 μL supernatant were mixed with 3 mL Rotiszint^®^eco plus scintillation cocktail (Carl Roth, Karlsruhe, Germany) and radioactivity was determined by scintillation counting.

### Quantification of Sulfate

Sulfate contents were analyzed by anion exchange chromatography as described by Herschbach et al. (2000). For this purpose, 20–30 mg homogenized frozen leaf tissue were added to a mixture of ∼20 mg of pre-washed polyvinylpolypyrrolidone (PVP 6755, Sigma-Aldrich, Steinheim, Germany) and 1 mL distilled water previously incubated overnight at 4°C and suspended by shaking. Samples were incubated for 1 h at 4°C and afterward boiled for 15 min at 95°C in a water bath (Julabo, Seelbach, Germany). After centrifugation for 15 min at 15,000 × *g* and 4°C, supernatants were transferred into new tubes. Aliquots of 150 μL were pipetted into glass vials (1.5 mL, 32 × 11.6 mm; VWR International, Darmstadt, Germany) and subjected to anion analysis using an automatic ion analyser (DX 120, Dionex, Sunnyvale, CA, United States), equipped with an IonPac^TM^ column (AS9-SC, 4 × 250 mm; Dionex, Thermo Fisher, Waltham, MA, United States). Anions were eluted with a mixture of 2.0 mM Na_2_CO_3_ and 0.75 mM NaHCO_3_. Sulfate was detected by a conductivity detector module (Dionex, Sunnyvale, CA, United States).

### Metabolite Measurements

#### Metabolite Measurement by GC-MS

Aliquots of 50–80 mg frozen leaf material were extracted with a mixture of chloroform-methanol-water for analysis of sugars, organic acids and amino acids by gas chromatography-mass-spectrometry (GC-MS) according to Fiehn et al. (2000), using a 7200 GC-QTOF (Agilent, Milford, CT, United States). Peak integration was conducted with MassHunter Software from Agilent. For relative quantification, metabolite peak areas were normalized to the amount of extracted plant material and the peak area of the internal standard ribitol added to the extraction solution.

#### Met and Ser Quantification by UHPLC-DAD

Extracts, as prepared for GC-MS analysis (described above), were dried and resolved in 50 μL HCl (0.1 M). Aliquots of 10 μL were derivatized with AccQ-Tag Ultra Reagent Powder (Waters, Milford, CT, United States) according to the manufacturer’s instructions. Derivatized samples were separated by liquid chromatography and amino acids were detected at 260 nm, using a 1290 UHPLC system coupled to a diode array detector (Agilent, Santa Clara, CA, United States) according to Rademacher et al. (2016). Peaks were integrated using the Chemstation software from Agilent and quantified by external standards after normalization to the internal standard norvaline, added prior to derivatization and the amount of extracted plant material.

### Statistical Analysis of Metabolite and Metabolite Flux Data

**FIGURE 1:**
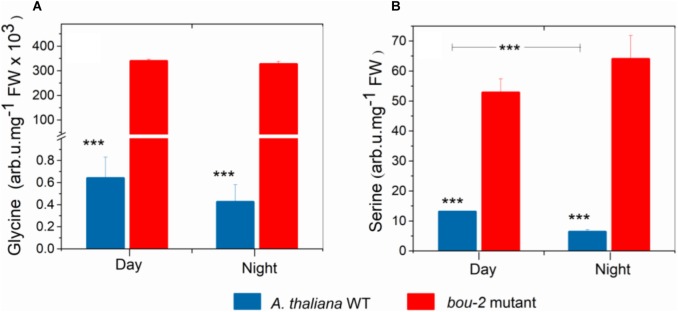
Content of Glycine **(A)**, Serine **(B)** in leaves of *A. thaliana* WT and the *bou-2* mutant at day and night. Plants were grown at elevated (3000 ppm) CO_2_ and harvested after 24h at ambient CO_2_ ca. 10 min after light onset and 3 h before light onset. Asterisks indicate significant differences as determined by Student’s *t*-test (^∗^*P* < 0.05, ^∗∗^*P* < 0.01, ^∗∗∗^*P* < 0.001). All values are means ± standard deviation of 3 replicates.

Metabolite and [^35^S] flux data were statistically analyzed using the software package SigmaPlot V. 11.0 (Systat, Erkrath, Germany). Prior to the analysis of variance, normality of the data was tested with the Shapiro–Wilk test. To determine significant differences between the WT and the *bou-2* mutant within the same time point and between day and night within the same plant genotype, Student’s *t*-test was performed. Sulfate uptake and flux into sulfur pools were determined with 4, metabolite contents with 3 replicates. All data shown represent means + SD. Principal component analysis (PCA, Supplementary Figure S1) was produced with function prcomp (%) implemented in the R statistics software. Adapted venn diagram (Supplementary Figure S1) was calculated with an in-house R script. The evaluation of data in Venn diagrams and PCA plots are provided with Supplementary Figure S1. The graphic work was performed either in OriginPro V.9.1. (Additive, Friedrichsdorf, Germany) (Figures 1, 6 and Supplementary Figure S3) or R statistics software (R version 3.3.0 provided by the CRAN project^1^).

### Next Generation Sequencing of RNA

#### RNA Extraction, Preparation, and Sequencing of Illumina Libraries

Whole rosettes were harvested and immediately frozen in liquid nitrogen. RNA was isolated from ground tissue using the RNeasy Plant Mini Kit (QIAGEN, Hilden, Germany). Residues of DNA were removed with DNase (New England Biolabs, Ipswitch, MA, United States). RNA integrity, sequencing library, and fragment size were analyzed on a 2100 Bioanalyzer (Agilent Technologies, Santa Clara, CA, United States). Libraries were prepared using the TruSeq RNA Sample Prep Kit v2 (Illumina, San Diego, CA, United States) and quantified with a Qubit 2.0 (Invitrogen, Waltham, MA, United States). Samples were multiplexed with 12 libraries per lane and sequenced in paired-end mode (Rapid Run, 150 bp read length) on an Illumina HiSeq 3000 platform, yielding on average ∼26 million reads per library.

#### Read Mapping and mRNA-Seq Data Analysis

After successful quality control with the Fast QC software^2^ (v0.11.5), Illumina reads were quantified by mapping against the *Arabidopsis* TAIR10 reference transcriptome including primary and secondary transcripts^3^ using Kallisto v 0.43.0 (Bray et al., 2016) in default mode with 30 times bootstrapping for sleuth. Kallisto provides normalized gene expression in transcripts per million (tpm). Primary transcripts for heat maps were chosen based on highest average tpm in the WT.

Wald test implemented in sleuth v0.28.1 (Pimentel et al., 2016) was employed to test for differential gene expression between WT and *bou-2* at day and night or between day and night within each genotype. If not indicated otherwise, *p*-values were corrected for multiple sampling by Benjamini–Hochberg correction (Benjamini and Hochberg, 1995) as implemented in sleuth and an alpha of 0.01 was chosen. Log_2_ expression ratios between genotypes or time points were calculated after addition of a pseudo-count of 1 to prevent infinite values.

## Results

### Consequences of Reduced GDC Activity and Day/Night on Gly and Ser Synthesis

**FIGURE 2:**
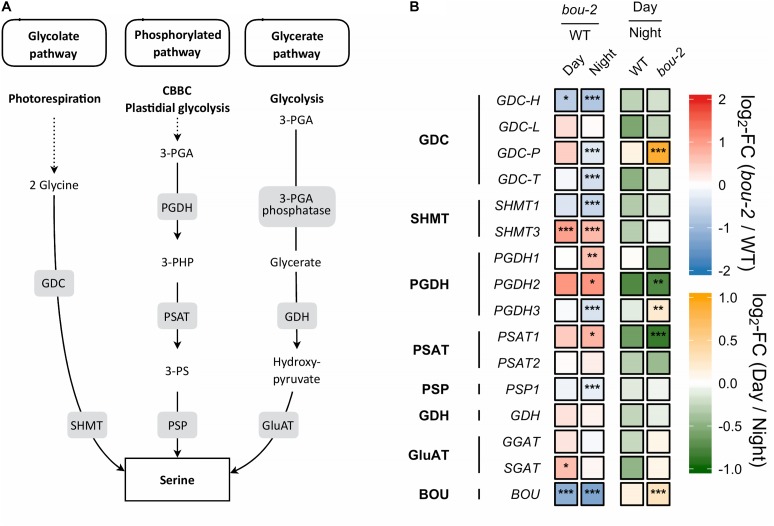
Photorespiratory and non-photorespiratory pathways of serine biosynthesis. **(A)** Schematic presentation of three pathways of serine biosynthesis (adapted from Ros et al., 2013) **(B)** transcript levels (*n* = 3) of genes involved in pathways depicted in panel **A**. Left two tiles of each gene represent the log2-fold change (FC) between the *bou-2* mutant and WT plants under both day (D) and night (N) with false color code ranging from red (higher in *bou-2*) to blue (higher in WT). Right two tiles represent the log2-fold change (FC) between day and night within WT plants and *bou-2* mutant with false color code ranging from green (higher at night) to yellow (higher at day). Asterisks indicate differential gene expression as determined by Sleuth (^∗^FDR < 0.05, ^∗∗^FDR < 0.01, and ^∗∗∗^FDR < 0.001). Levels of BOU (A BOUT DE SOUFFLE) shown for reference. GDC, glycine decarboxylase; SHMT, serine hydroxymethyltransferase; PGDH, phosphoglycerate dehydrogenase; PSAT, 3-phosphoserine aminotransferase; PSP, 3-phosphoserine phosphatase; GDH, glycerate dehydrogenase; GluAT, hydroxypyruvate aminotransferase.

**FIGURE 3:**
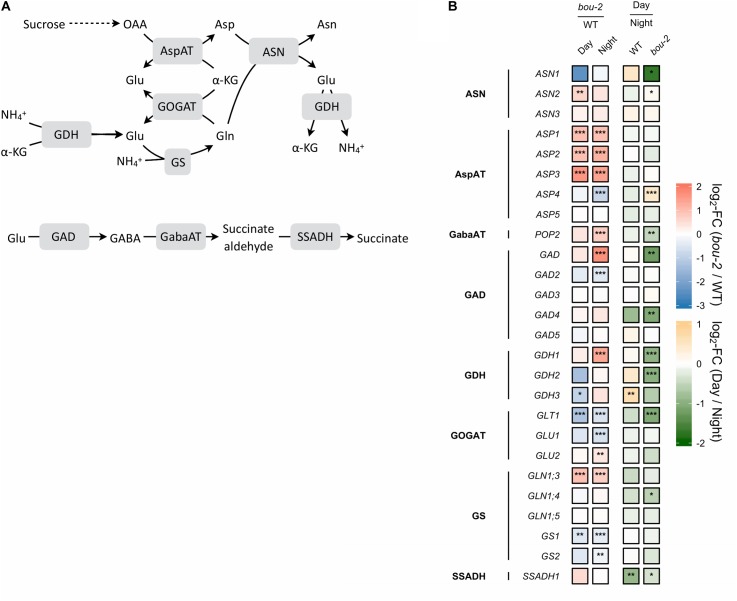
Nitrogen assimilation and the GABA shunt. **(A)** Schematic presentation of the pathways; **(B)** Transcript levels (*n* = 3) of genes encoding for enzymes highlighted in gray boxes in panel **A**. Left two tiles of gene represent the log2-fold change (FC) between the *bou-2* mutant and WT plants under both day (D) and night (N) with false color code ranging from red (higher in *bou-2*) to blue (higher in WT). Right two tiles represent the log2-fold change (FC) between day and night within WT plants and the *bou-2* mutant with false color code ranging from green (higher at night) to yellow (higher at day). Asterisks indicate differential gene expression as determined by Sleuth (^∗^FDR < 0.05, ^∗∗^FDR < 0.01, and ^∗∗∗^FDR < 0.001). AspAT, aspartate aminotransferase; ASN, asparagine synthetase; GABA, gamma-Aminobutyric acid; GabaAT, GABA aminotransferase; GDH, glutamate dehydrogenase; GOGAT, glutamate synthase; GS, glutamine synthase; SSADH, succinate semialdehyde dehydrogenase.

Despite pre-cultivation at elevated CO_2_ to suppress photorespiratory Gly production, leaves of the *bou-2* mutant with impaired GDC activity (Eisenhut et al., 2013) had accumulated large amounts of Gly after 24 h growth at ambient CO_2_ at both, day and night (Figure 1A). Leaves of the *bou*-2 mutant also contained higher levels of Ser at both time points, although Ser synthesis via SHMT is downstream of GDC (Figure 1B). In leaves of the WT, but not of the *bou-2* mutant, Ser levels were reduced in the night compared to day (Figure 1B). Transcript level for three of the four *GDC* isoforms (namely GDC-H, -P and -T) were significantly down-regulated in *bou-2* compared to the WT at night (Figure 2). *SHMT3* gene was up-regulated at day and night, while *SHMT1* was down-regulated at the night in the *bou-2* mutant (Figure 2). In addition, transcript abundances of genes encoding for one or multiple isoforms of seven key enzymes and transporters of photorespiration (*GOX1*, glycolate oxidase; *PLGG1*, plastidic glycolate glycerate transporter; *HPR1*, hydroxypyruvate reductase; *GLYK*, glycerate kinase) (Supplementary Figure S2) as well as the associated nitrogen assimilation (major isoforms of Fd-GOGAT and GS) were reduced in the *bou-2* mutant (Figure 3). Three genes encoding photorespiratory enzymes exhibited elevated transcript abundances in *bou-2*, including the major isoform of Ser-glutamate amino transferase (*SGAT*), *SHMT3* and catalase 1 (*CAT1*) and 3 (*CAT3*), but not *CAT2*, which is the primary isoform in WT plants (Supplementary Figure S2). These data indicated that the high level of Ser in leaves of the *bou-2* mutant cannot be explained by transcript abundances of genes of the photorespiratory pathway, and other processes including post-transcriptional and post-translational modifications cannot be excluded.

**FIGURE 4:**
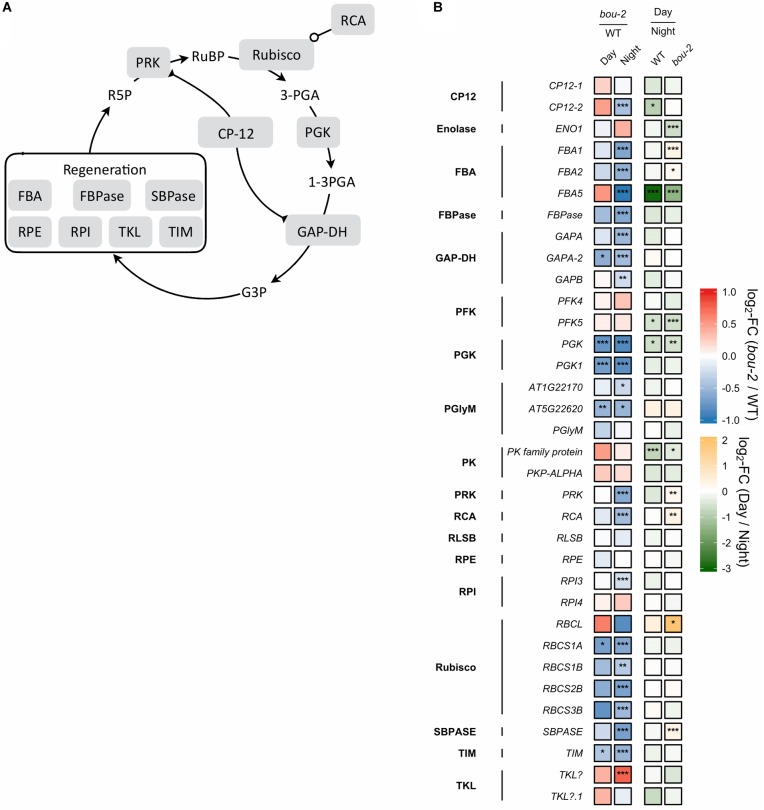
**(A)** Schematic presentation of the Calvin–Benson–Bassham cycle (CBBC). **(B)** Transcript levels (*n* = 3) of genes involved in CBBC. Left two tiles of each gene represent the log2-fold change (FC) between the *bou-2* mutant and WT plants under both day (D) and night (N) with false color code ranging from red (higher in *bou-2*) to blue (higher in WT). Right two tiles represent the log2-fold change (FC) between day and night within WT plants and *bou-2* mutant with false color code ranging from green (higher at night) to yellow (higher at day). Asterisks indicate differential gene expression as determined by Sleuth (^∗^FDR < 0.05, ^∗∗^FDR < 0.01, and ^∗∗∗^FDR < 0.001). CP12, CP12 domain-containing protein; FBPase, fructose bisphosphatase; GAP-DH, glyceraldehyde 3-phosphate dehydrogenase; PGK, phosphoglycerate kinase; PRK, phosphoribulokinase; RCA, rubisco activase; RPE, ribulose-phosphate 3-epimerase; RPI, ribose-5-phosphate isomerase; Rubisco, ribulose-bisphosphate carboxylase; SBPASE, sedoheptulose-1,7-bisphosphatase; TKL, transketolase.

Gene expression of the other two pathways of Ser biosynthesis in the leaves were not as strongly altered in the *bou-2* mutant as gene expression of the photorespiratory pathway, with the glycerate pathway being the least affected. In the phosphorylated pathway of Ser synthesis, 3-phosphoglycerate (3-PGA) is converted to 3-phosphohydroxypyruvate (3-PHP) by 3-PGA dehydrogenase (PGDH) and further to phosphoserine by 3-PS aminotransferase (PSAT). While transcript abundances of *PGDH1*, *PGDH2*, and *PSAT1* encoding these enzymes were higher in the *bou-2* mutant at night, abundance of 3-phosphoserine phosphatase (*PSP1*), encoding the protein catalyzing the final step of Ser synthesis, was depleted in the *bou-2* mutant compared to the WT (Figure 2). This plastidial pathway is dependent on the precursor molecule 3-PGA sourcing from the Calvin–Benson–Bassham Cycle (CBBC) or plastidal glycolysis. Out of 33 genes encoding for enzymes of these pathways 22 were downregulated at one or both time points in *bou-2*, including most subunits of rubisco (Figure 4).

**FIGURE 5:**
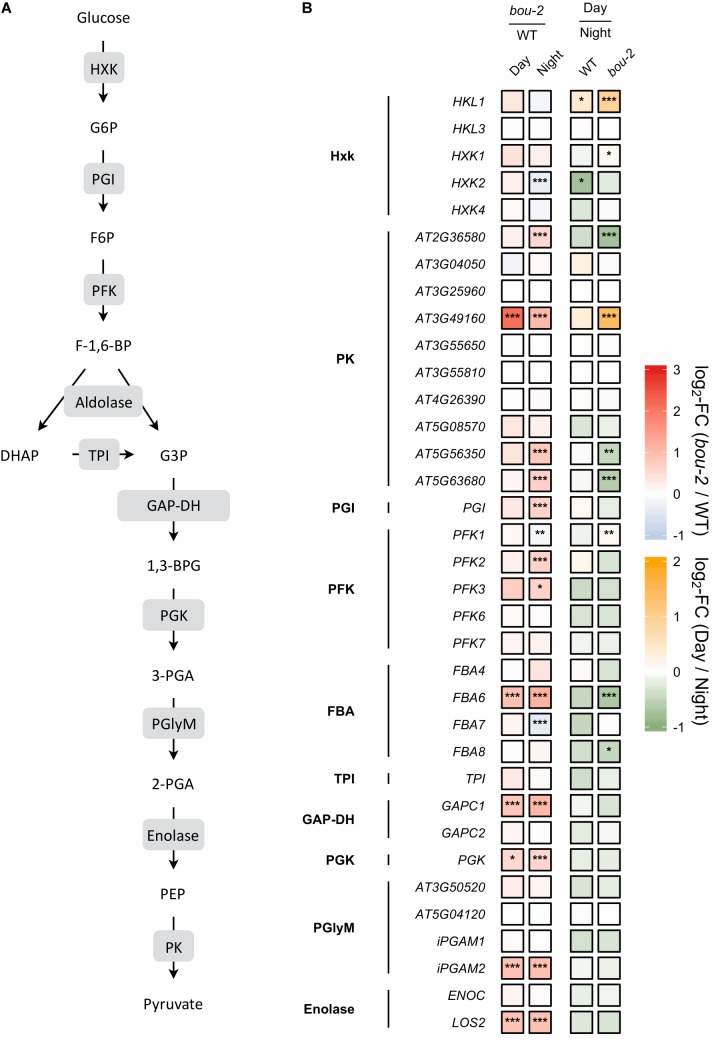
**(A)** Schematic presentation of glycolysis. **(B)** Transcript levels of genes encoding for enzymes highlighted in gray boxes in panel **A**. Left two tiles of each gene represent the log2-fold change (FC) between the *bou-2* mutant and WT plants under both day (D) and night (N) with false color code ranging from red (higher in *bou-2*) to blue (higher in WT). Right two tiles represent the log2-fold change (FC) between day and night within WT plants and the *bou-2* mutant with false color code ranging from green (higher at night) to yellow (higher at day). Asterisks indicate differential gene expression as determined by Sleuth (^∗^FDR < 0.05, ^∗∗^FDR < 0.01, and ^∗∗∗^FDR < 0.001). HXK, hexokinase; PGI, phosphoglucose isomerase; PFK, phosphofructokinase; FBA, fructose-bisphosphate aldolase; TPI, triosephosphate isomerase; GAP-DH, glyceraldehyde-3-phosphate dehydrogenase; PGK, phosphoglycerate kinase; PGlyM, phosphoglycerate/bisphosphoglycerate mutase; PK, Pyruvate kinase.

The glycerate route of Ser synthesis is fed by 3-PGA produced by cytosolic glycolysis. Ten genes encoding for enzymes of cytosolic glycolysis, including enolase, FBA, GAP-DH, PFK, phosphoglycerate mutase and pyruvate kinase were more abundant in leaves of the *bou-2* mutant (Figure 5). Apparently, also transcript abundances of the two alternative pathways of Ser synthesis cannot explain Ser accumulation in the *bou-2* mutant. Since metabolite accumulation can not only be a consequence of enhanced production, but also of reduced consumption, we assessed the use of Gly and Ser in sulfur assimilation and the flux of S into major sulfur pools, namely GSH, GLS and protein.

### Consequences of Reduced GDC Activity and Day/Night on Sulfur Metabolism

#### Reduced GDC Activity Mediated a Decline in Sulfur Flux Into Major Sulfur Pools in the *bou-2* Mutant

**FIGURE 6:**
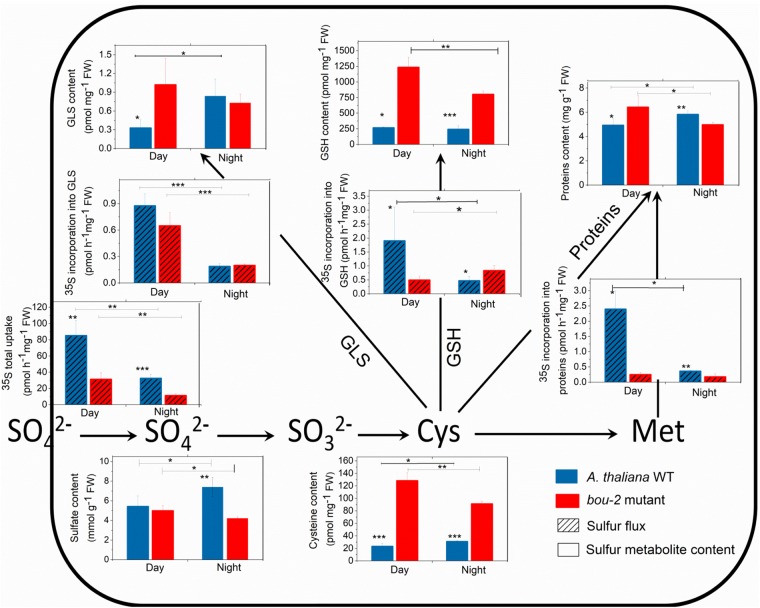
Foliar sulfur metabolite content and sulfur fluxes in the *A. thaliana* WT and the *bou-2* mutant during day and night. Asterisks indicate significant differences as determined by Student’s *t*-test (^∗^*P* < 0.05, ^∗∗^*P* < 0.01, and ^∗∗∗^*P* < 0.001). All values are means ± standard deviation of 4 replicates.

Sulfate uptake into the leaves was reduced during night compared to day and was lower in *bou-2* than in WT leaves at both, day and night (Figure 6). These significant differences were observed despite similar or even enhanced (WT, night) pool sizes of sulfate (Figure 6). In WT leaves, reduced sulfate uptake in the night also reduced the flux of sulfate into GSH, protein, and GLS. Again, this was observed at similar pool sizes of Cys, GSH, protein and GLS. During day, the flux of sulfate into the GSH, protein, and GLS pools was strongly reduced in *bou-2* compared to WT leaves. However, this was accompanied by a strongly enhanced Cys and GSH pool in *bou-2*, whereas the protein and GLS pools were only slightly affected. During the night the flux of sulfate into GLS, but not into GSH or protein was reduced in *bou-2* mutant (Figure 6). Pool sizes of Cys and GSH in *bou-2* leaves were slightly reduced in the night compared to the day but still remained significantly higher than in WT controls (Figure 6). These results suggest that a reduced flux of sulfur into GSH and protein may have contributed to the observed accumulation of Ser and Gly in *bou-2* leaves. The high pool sizes of Cys and GSH in *bou-2* at both day and night cannot be attributed to the shift from elevated to ambient CO_2_, because thiol levels were also enhanced in leaves of *bou-2* compared to the WT at similar protein content, when plants were harvested before this shift (Supplementary Figure S3). Although the sulfur flux in the *bou-2* mutant is reduced, the high pool size of GSH and Cys is presumably due to their lower consumption in anabolic and/or catabolic processes.

#### Reduced GDC Activity in the *bou-2* Mutant Mediated Deregulation of Genes of Sulfur Reduction and Assimilation

**FIGURE 7:**
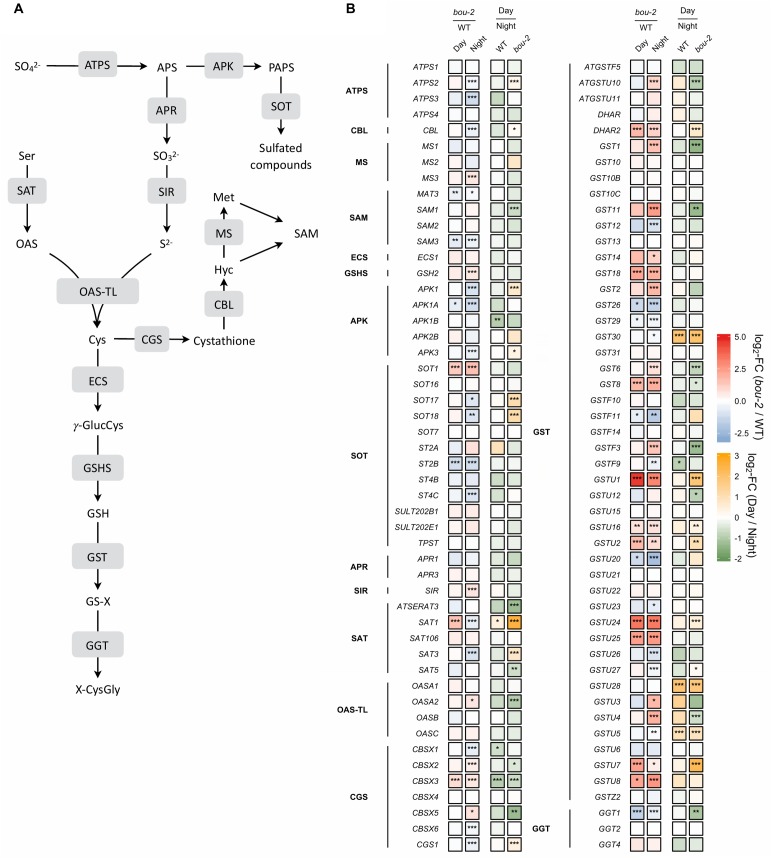
Schematic presentation **(A)** and transcript levels (**B**, *n* = 3) of genes involved in leaf sulfur metabolism. Left two tiles of each gene represent the log2- fold change (FC) between the *bou-2* mutant and the WT plants at both day (D) and night (N) with false color code ranging from red (higher in *bou-2*) to blue (higher in WT). Right two tiles represent the log2-fold change (FC) between day and night within the WT plants and the *bou-2* mutant with false color code ranging from green (higher at night) to yellow (higher at day). Asterisks indicate differential gene expression as determined by Sleuth (^∗^FDR < 0.05, ^∗∗^FDR < 0.01, and ^∗∗∗^FDR < 0.001). APS, adenosine 5′- phosphosulfate; Cys, cysteine; Cyst, cystathionine; Glu, glutamate; γ-GluCys, γ-glutamylcysteine; GSH, glutathione; GS-X, glutathione conjugate; Hcy, homocysteine; Met, methionine; OAS, O-acetylserine; OPH, O-phosphohomoserine; PAPS, 3′-phosphoadenosine 5′- phosphosulfate; ROH, hydroxylated precursor; SAH, S-adenosylhomocysteine; SAM, S-adenosylmethionine; Ser, serine; Thr, threonine; X-CysGly, cysteinylglycine conjugate. Enzymes: ATPS, ATP sulfurylase; APK, APS kinase; SOT, sulfotransferase; APR, APS reductase; SIR, sulfite reductase; SAT, serine acetyltransferase; OAS-TL, OAS(thiol)lyase; CGS, cystathionine gamma-synthase; TS, threonine synthase; CBL, cystathionine beta-lyase; MS, methionine synthase; SAM, S-adenosylmethionine synthetase; ECS, gamma -glutamylcysteine synthetase; GSHS, glutathione synthetase; GST, glutathione-S-transferase; GGT, gamma-glutamyltransferase.

In sulfur metabolism depicted in Figure 7, more genes were significantly changed during the night than during the day in *bou-2* compared to WT leaves. The expression of ATP sulfurylase that catalyses the activation of sulfate at the very first step of assimilatory sulfate reduction was significantly lower in the mutant at night compared to the WT (Figure 7). The cytosolic *ATPS* gene (*ATPS2*) showed a significant increase in the transcript level in *bou-2* at day compared to night. This is in agreement with strongly reduced ^35^S uptake by the mutant compared to the WT and by both the WT and the mutant at night compared to day (Figure 7). The *APR1* and *APR3* genes, which are considered the key regulatory enzyme of sulfate assimilation (Koprivova et al., 2000), did not show any significant change, neither between WT and *bou-2*, nor between day and night (Figure 7). However, transcript abundance of sulfite reductase (SiR) that converts sulfite into sulfide was significantly higher in leaves of the mutant at night compared to the WT (Figure 7). Chloroplastic *SAT* (*SAT1*) was the only isoform of SAT that showed significant upregulation in the mutant during day compared to the WT (Figure 7). The transcript level of *SAT1* together with mitochondrial *SAT3* significantly decreased at night in the mutant compared to the WT. Nevertheless, both genes were significantly upregulated in the mutant at the day compared to the night, while cytosolic *SAT5* was slightly but significantly downregulated (Figure 7). In the mutant, the cytosolic *OAS-TL* gene (*OASA*) showed a significantly higher transcript level in the mutant at night compared to the day. Thus, higher Cys contents in the mutant at night and day compared to the WT cannot be explained by a change in enzyme abundances based on transcriptional changes of *SAT* and *OAS-TL* genes in the mutant. Apparently, the enhanced Cys content in the *bou-2* mutant is not due to transcriptional changes of genes of sulfate reduction and assimilation and presumably a result of post-transcriptional and/or post-translational regulation.

#### In the *bou-2* Mutant, Cys Is Largely Used for GSH While Its Use for Met and GLS Synthesis Is Reduced

In plants, Cys is used for protein synthesis either directly or after its metabolic conversion to methionine *via* cystathionine. In addition, Cys is used for GSH and GLS synthesis. In the present study, expression of the gene encoding γ-glutamylcysteine synthetase (γ–ECS), the first step of GSH synthesis, was not significantly different between day and night for either WT or *bou-2* leaves (Figure 7). In the WT, significant differences between day and night were not observed also in the transcript abundance of the gene encoding glutathione synthetase (*GSH2*) that produces GSH from γ-glutamylcysteine and glycine. However, at night the *GSH2* transcript level was significantly higher in the mutant compared to the WT. Upregulation of *GSH2* together with the significantly higher transcript level of *SiR* and *OASA* genes could explain the higher flux into GSH in the mutant at night (Figure 6). However, the almost five-fold higher content of GSH at day and the three-fold higher level at night in mutant compared to WT leaves as well as differences in the GSH pool of the mutant between night and day (Figure 6), could not completely be explained by changes in the transcript abundance of *GSH2*.

**FIGURE 8:**
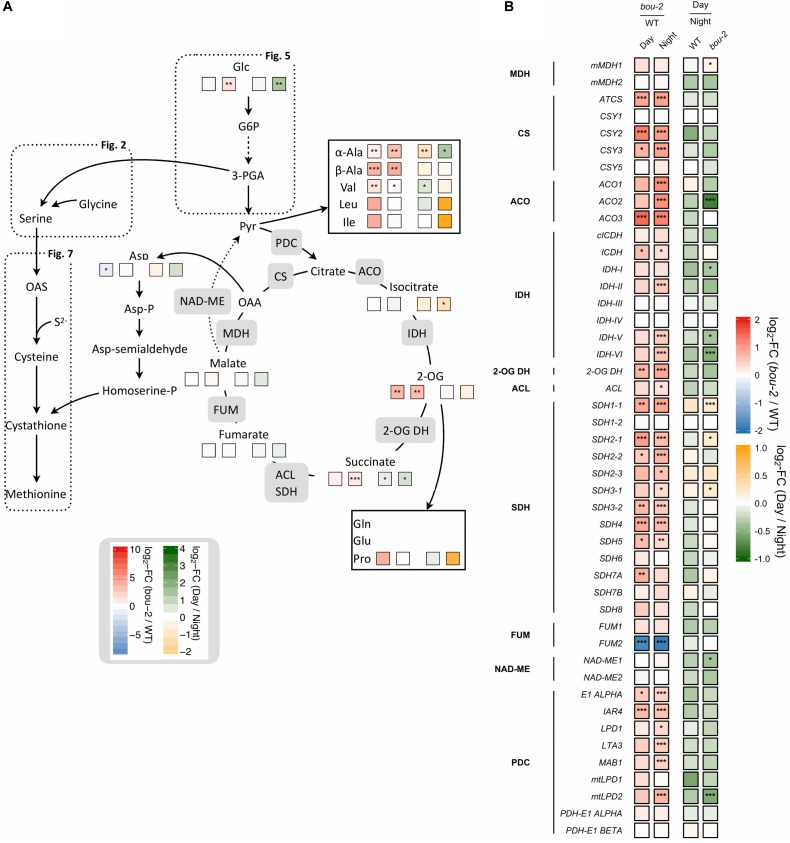
Cross-talk of sulfur with nitrogen assimilation and catabolic carbon metabolism. **(A)** Schematic presentation of amino acids and other metabolites which closely interact with sulfur metabolism in serine and cysteine biosynthesis analyzed in leaves of the *A. thaliana* WT and the *bou-2* mutant at day and night exposure (*n* = 4). **(B)** Transcript levels (*n* = 3) of genes encoding for enzymes of the tricarboxylic acid (TCA) cycle. Left two tiles represent the log2-fold change (FC) between the *bou-2* mutant and the WT plants at both day (D) and night (N) exposure with false color code ranging from red (higher in *bou-2*) to blue (higher in WT). Right two tiles represent the log2-fold change (FC) between day and night exposure within the WT plants and *bou-2* mutant with false color code ranging from green (higher at night) to yellow (higher at day). Asterisks indicate significantly different metabolite levels **(A)** as determined by Student’s *t*-test (^∗^*p* < 0.05, ^∗∗^*p* < 0.01, and ^∗∗∗^*p* < 0.00) or differential gene expression **(B)** as determined by Sleuth (^∗^FDR < 0.05, ^∗∗^FDR < 0.01, and ^∗∗∗^FDR < 0.001). 1, malate dehydrogenase (MDH); 2, citrate synthase (CSY); 3, aconitase (ACO); 4, isocitrate dehydrogenase (IDH); 5, 2-OG dehydrogenase (2OG DH); 6, Succinate-CoA ligase (ACL); 7, succinate dehydrogenase (SDH); 8, fumarase (FUM); 9, NAD-malic enzyme (NAD-ME); 10, pyruvate dehydrogenase.

In the present study, it seems that Cys consumption for Met synthesis was reduced (Figures 7, 8A). Two first enzymes of Met biosynthesis, cystathionine γ-synthase (*CGS1*) and cystathionine β-lyase (*CBL*) did not show considerable differences in the transcript level between WT and *bou-2* at day, but exhibited a significant decrease in the mutant at night compared to the WT (Figure 7). Still the chloroplastic methionine synthase (*MS3*) transcript level was significantly higher in the mutant at night (Figure 7). However, it seems that *MS3* is not responsible for a major part of Met synthesis, as the Met content was significantly lower in the mutant at day and night compared to the WT. These differences in Met contents are consistent with significantly lower transcript levels of *CGS* and *CBL* genes in the mutant (Figure 7 and Table 1).

**Table 1:** Metabolite contents present in arbitrary units (arb.u. mg^-1^ FW) in leaves of *Arabidopsis thaliana* WT and the *bou-2* mutant at day and night.

	Metabolites	Day		Night	
			
		Col-0	BOU	Col-0	BOU
		Mean *SD*	Mean *SD*	Mean *SD*	Mean *SD*
Sugars	Fructose	2099.02 ± 251.05a*	3128.45 ± 830.72*	1301.38 ± 229.22^a^	7401.39 ± 1774.24^b^
	Glucose	3587.00 ± 1178.37	2804.70 ± 428.45**	2879.78 ± 400.50^a^	8374.86 ± 1683.53^b^
	Maltose	189.11 ± 67.26***	108.42 ± 10.53 ***	648.76 ± 13.94^a^	267.89 ± 11.22^b^
	Manose	45.86 ± 11.28	34.38 ± 2.27*	61.51 ± 4.59^a^	46.14 ± 5.28^b^
	Raffinose	152.52 ± 19.61^a^	0.37 ± 0.30^b^	237.985 ± 86.18^a^	0.78 ± 0.74^b^
	Sorbitol	43.79 ± 9.81^a^**	29.55 ± 3.92^b^***	17.95 ± 2.31^a^	11.59 ± 0.48^b^
	Sucrose	27661.97 ± 3859.03	25636.56 ± 1699.43*	28159.45 ± 1150.18^a^	19927.22 ± 2573.64^b^
	Xylose	88.33 ± 9.402	94.13 ± 10.99	92.26 ± 1.66	99.25 ± 7.33
Acids involved in TCA	Alpha-ketoglutarate	24.95 ± 9.46^a^	304.22 ± 56.91^b*^	19.49 ± 0.90^a^	200.92 ± 21.30^b^
	Fumaric acid	56114.14 ± 3677.00	40858.41 ± 9527.63	54647.13 ± 5059.05	51553.88 ± 2507.11
	(Iso)citric acid	1596.74 ± 664.04	1429.45 ± 387.71*	949.93 ± 272.408	580.83 ± 357.611
	Malic acid	4412.55 ± 651.93	4701.32 ± 546.39*	4509.1 ± 451.8^a^	6160.18 ± 297.17^b^
	Proline	104.85 ± 20.13*	212.29 ± 64.24	57.74 ± 2.38	111.34 ± 36.35
	Succinic acid	349.30 ± 55.03^a^	625.65 ± 148.07^b*^	436.55 ± 20.61^a^	901.08 ± 24.12^b^
Secondary metabolites	Glycolic acid	31.38 ± 2.88^a^**	40.04 ± 2.23^b^***	19.96 ± 0.62^a^	22.88 ± 0.28^b^
	Shikimate	340.53 ± 6.73^a*^	192.09 ± 22.094^b*^	411.33 ± 26.960^a^	267.06 ± 37.963^b^
	Quinic acid	1.75 ± 0.17**	1.51 ± 0.072***	2.54 ± 0.09^a^	2.86 ± 0.03^b^
Amino acids	Alfa alanine	1212.82 ± 203.49^a^**	1910.62 ± 36.03^b**^	564.43 ± 30.08^a^	5831.68 ± 1123.55^b^
	Aspartic acid	20.54 ± 3.24^a*^	8.31 ± 0.39^b^***	12.22 ± 2.33^a^	19.57 ± 2.26^b^
	Beta-alanine	34.30 ± 0.68^a^	673.08 ± 98.08^b^	35.54 ± 2.31^a^	632.54 ± 100.52^b^
	GABA	2774.76 ± 99.02^a*^	4649.08 ± 367.88^b^	2331.90 ± 182.87^a^	4597.98 ± 752.32^b^
	Glyceric acid	153.36 ± 32.22^a*^	310.06 ± 21.39^b^***	90.48 ± 8.24^a^	133.94 ± 5.85^b^
	Glycine	637.59 ± 192.41^a^	338399.99 ± 8441.71^b^	423.50 ± 157.21^a^	325863.04 ± 11640.42^b^
	(Iso)leucine	11.32 ± 0.88^a^	78.62 ± 25.44^b*^	12.00 ± 1.97^a^	16.75 ± 0.53^b^
	Leucine	11.24 ± 0.24^a^	73.48 ± 16.99^b**^	12.97 ± 2.36	15.84 ± 1.61
	Serine	13.01 ± 0.33^a***^	52.78 ± 4.64^b^	6.28 ± 0.76^a^	63.91 ± 7.93^b^
	Threonine	7.52 ± 1.09^a***^	41.38 ± 8.22^b*^	2.01 ± 0.38^a^	16.17 ± 5.34^b^
	Valine	356.05 ± 101.33^a^**	1169.77 ± 19.021^b^***	631.58 ± 8.99^a^	717.51 ± 37.84^b^
Sugar alcohols	Methionine	6.374 ± 0.761^a^	3.055 ± 0.679^b^	4.950 ± 1.162^a^	1.797 ± 0.955^b^
	Glycerol	1728.74 ± 83.86^a^	2065.05 ± 40.31^b*^	1816.16 ± 111.70^a^	3055.49 ± 415.78^b^
Organic acids	Mannitol	98.69 ± 15.51	88.92 ± 5.43***	70.20 ± 17.21	42.33 ± 7.98
	Gluconic acid	5.89 ± 0.34^a*^	9.01 ± 1.77^b^	4.39 ± 0.63	5.84 ± 0.95
	Hydroxyglutarat	28.58 ± 6.03^a^	55.15 ± 1.38^b^***	40.25 ± 7.89	36.88 ± 1.94
	Maleic acid	57.91 ± 11.28	51.36 ± 7.60	54.06 ± 4.93	48.72 ± 7.45
	Malonic acid	0.86 ± 0.17^a***^	3.85 ± 0.88^b^	2.61 ± 0.14	3.88 ± 0.78
	Myoinositol	2633.09 ± 246.98^a^**	1085.52 ± 120.82^b**^	4197.10 ± 364.20^a^	1742.63 ± 211.04^b^


*Plants were grown at elevated (3000 ppm) CO_2_ and harvested after 24 h of ambient CO_2_ level ca. 10 min after light onset and 3 h before light onset. Small letters indicate significant differences between different plant types within same treatment (*P* < 0.05). Asterisks indicate significant differences between day and night within same plant type (^∗^*P* < 0.05, ^∗∗^*P* < 0.01, and ^∗∗∗^*P* < 0.001). All values are means ± standard deviation of 3 replicates.*

Apart from sulfur consumption for GSH and Met, part of the sulfur taken up is used for the synthesis of secondary metabolites such as GLS. Transcript levels of the cytosolic APS kinase isoform *APK1* were significantly downregulated in the *bou-2* mutant at day compared to the WT, and together with *APK3* also at night. Furthermore, both isoforms were significantly upregulated in the *bou-2* mutant at day compared to night (Figure 7). The sulfotransferase genes *SOT17* and *SOT18*, responsible for transferring the sulfate moiety from PAPS to the preferentially Met-derived desulfoglucosinolate (dsGS) (Piotrowski et al., 2004), showed a low transcript level in the mutant at night compared to the WT. However, this decrease did not affect the S flux into the GLS pool (Figure 6). Rather, the increased flux of S into GLS in the mutant at day compared to night might be a result of significantly higher transcript abundances of the *APK1*, *APK3*, *SOT17*, and *SOT18* genes (Figures 6, 7).

#### High Cys and GSH Contents Are Not a Result of Reduced GSH and/or Protein Degradation

The transcript level of the *GGT1* gene, encoding γ-glutamyltransferase, which catalyses the first step of GSH degradation, is significantly lower in the mutant at day and night compared to the WT at night (Figure 7). This result suggests that the high amount of Cys in the mutant is not a consequence of GSH degradation. This view is supported by high GSH contents of the mutant at day and night compared to WT leaves (Figure 6). The high Cys and GSH contents in the mutant also cannot be attributed to protein degradation, because protein contents in WT and mutant leaves are almost same both, at day and night (Figure 6).

### Consequences of Reduced GDC Activity and Day/Night for the Metabolic Crosstalk of Sulfur With Carbon and Nitrogen Metabolism

#### Reduced GDC Activity in the *bou-2* Mutant Mediated Downregulation of Genes of Calvin–Benson–Bassham Cycle (CBBC) and Accumulation of Sugars

Reduced GDC activity in the *bou-2* mutant excessively influenced gene transcript levels of most enzymes of the CBBC. 18 of 33 examined genes were downregulated in the mutant at both day and night compared to the WT (Figure 4). The *GAPA-2* gene encoding glyceraldehyde-3-phosphate dehydrogenase together with *PGK, PGK1* of phosphoglycerate kinase (PGK) and genes of Rubisco subunits were significantly downregulated at day and night (Figure 4). As a consequence, foliar sugar accumulation was affected (Table 1). During day a slight increase in the fructose content at the expense of glucose was observed in leaves of the mutant. However, consequences of the mutation for sugar accumulation were much more pronounced at night. Fru and Glc accumulated 1.5 and 5.7 times more in the *bou-2* mutant compared to the WT at day and night, respectively. In addition, maltose content decreased in the mutant to 57% and 41% of the WT level, while raffinose contents declined to 0.2 and 0.3% of the WT content, both at day and night, respectively. In leaves of the WT and the *bou-2* mutant the contents of these sugars increased from day to night.

#### Reduced GDC Activity Mediated an Upregulation of Key Enzymes of the Glycolysis Pathway in the *bou-2* Mutant

In glycolysis, sugars are oxidized to generate energy, pyruvate and building blocks for anabolic processes (Plaxton, 1996). Reduced GDC activity affected 20 of 35 presented genes of this pathway in the mutant at day and night compared to the WT (Figure 5). In addition, most genes of this pathway were downregulated in WT and mutant leaves at day compared to night. Hexokinase (HXK) and ATP-dependent phosphofructokinase (PFK) are key regulatory enzymes that play important roles in sugar sensing and signaling (*PFK*: Plaxton, 1996; *HXK*: Jang et al., 1997). In the present study, transcript levels of *HXK*2 and *PFK1* were significantly lower, while the isoforms *PFK2* and *PFK3* exhibited significantly higher transcript levels in the mutant at night compared to the WT (Figure 5). Upstream of 3-PGA the isoform *FBA6* of fructose-bisphosphate aldolase (FBA) together with *GAPC1* encoding the glyceraldehyde-3-phosphate dehydrogenase (*GAP-DH)* and the *PGK* gene encoding phosphoglycerate kinase exhibited higher transcript abundances in the *bou-2* mutant at both, day and night compared to the WT (Figure 5). Furthermore, the gene isoforms *HKL1*, *FBA1*, *FBA2*, and *PKP4* showed higher transcript levels at day in mutant compared to night (Figure 5). Downstream of 3-PGA in the glycolysis pathway, pyruvate kinase (PK) is of major importance as a key regulatory step (Plaxton, 1996), catalyzing the conversion of phospho*enol*pyruvic acid (PEP) into ATP and pyruvate (Figure 5). Transcripts of four pyruvate kinase (PK) genes were significantly more abundant in the *bou-2* mutant at night compared to the WT (Figure 5). The upregulation of the key genes of the glycolysis pathway at night and day in the mutant provokes the hypothesis that this upregulation mediates the production of pyruvate used as C-skeleton for enhanced production of amino acids such as Cys, Ala, Val, Leu, and Ile and intermediates of the TCA cycle such as malate, succinate and α-ketoglutarate (2-OG) (Figure 8).

#### Reduced GDC Activity Mediated an Alternation of TCA Cycle Functions in the *bou-2* Mutant by Upregulation of Genes and Accumulation of Intermediates

Intermediates of the TCA cycle were affected in the *bou-2* mutant. The contents of malate, succinate and 2-OG increased in the mutant by 6, 44, and 92%, respectively, at day and by 27, 51, and 90%, respectively, at night (Figure 8A and Table 1). This accumulation may be attributed to a reduced use of TCA cycle intermediates in amino acid synthesis as indicated by reduced levels of Asp and Met at day and night (Figure 8A). On the other hand, Pro and Thr, also originating from TCA cycle intermediates, were increased in the mutant by 82 and 88%, respectively, for day and night (Figure 8A). Such an increase was also observed for a number of pyruvate derived amino acids, namely Ala, Val, Leu, and Ile (Figure 8A and Table 1), suggesting enhanced operation of glycolysis.

RNA-Seq results showed a significant upregulation of numerous genes of the TCA cycle in the mutant at day and night (Figure 8). All TCA cycle genes were downregulated at day compared to night in WT and mutant leaves except for three genes encoding mitochondrial succinate dehydrogenase, namely *SDH1-1*, *SDH2-1*, and *SHD3-1*. Transcript abundances of these genes were significantly upregulated at day in the mutant compared to WT leaves (Figure 8). Transcript levels of the peroxisomal citrate synthase genes *CSY2* and *CSY3*, involved in the sub-pathway of isocitrate synthesis from oxaloacetate (OAA) and acetyl-CoA, were two-fold higher in the mutant at day and night compared to the WT (Figure 8). Genes encoding for aconitase (ACO), which catalyzes the isomerization of citrate to isocitrate in the TCA cycle (Figure 8), showed upregulated transcript levels in the mutant at night (*ACO1* and *ACO2*) or day and night (*ACO3*) compared to the WT (Figure 8). The *ACO2* gene also showed significantly higher expression in the mutant at night compared to day (Figure 8). Upregulation of genes downstream of the sub-pathway of isocitrate synthesis coincided with a decrease in isocitrate content in the mutant at night (Figure 8A). However, higher accumulation of isocitrate was observed in the mutant at day compared to night (Table 1). This accumulation in the mutant could be a consequence of the downregulated transcript levels of genes encoding isocitrate dehydrogenase (IDH) subunits I, V and VI, which catalyze the oxidative transformation of isocitrate to 2-OG (Figure 8). However, as for isocitrate, also accumulation of 2-OG was observed in the mutant at day compared to night (Figure 8A and Supplementary Dataset S1). The enhanced 2-OG content in the mutant at day might originate from different processes. The major mitochondrial IDH subunits (*IDH-II, IDH-V IDH-VI*) showed significantly increased transcription at night in the *bou-2* mutant compared to the WT (Figure 8) that may result in enhanced 2-OG synthesis. Enhanced gene expression was also observed for succinate dehydrogenase (*SDH*) subunits that catalyze fumarate synthesis from succinate, specifically for *SDH1-1*, *SDH2-1*, *SDH2-2*, SDH3-2, *SDH4*, and *SDH5* genes, in leaves of the *bou-2* mutant at day and night compared to the WT (Figure 8). Also, genes encoding pyruvate dehydrogenase that transform pyruvate into acetyl-CoA were significantly upregulated in the *bou-2* mutant at day and night (Figure 8). Together with the high amount of pyruvate derived amino acids (Figure 8A and Table 1) this result indicates an enhanced foliar supply of pyruvate in the mutant for both, the reactions of the TCA cycle and the synthesis of pyruvate derived amino acids.

#### Reduced GDC Activity Mediated Downregulation of Genes of Nitrogen Assimilation

Photorespiration effects not only carbon but also nitrogen metabolism (Eisenhut et al., 2013, 2015). Among the genes encoding glutamine synthetase (GS), transcript abundance of *GS1* was downregulated in the mutant at day and together with *GS2* also at night (Figure 3) compared to the WT. Transcript level of *GLT1*, encoding glutamate synthase (Fd-GOGAT), showed significantly stronger downregulation in the mutant at day and together with *GLU1* at night compared to the WT (Figure 3). *GLU1* encodes a ferredoxin-dependent glutamate synthase. Different to *GLU1*, *GLU2* showed a significantly enhanced transcript level in the mutant at night. Further, the transcript abundance of *GLT1* was significantly downregulated in the mutant at day compared to night (Figure 3). The downregulation of genes of the nitrogen assimilation pathway (GS/GOGAT pathway) in the mutant compared to the WT might be due to reduced production of NH_4_^+^ during photorespiration at reduced GDC activity.

Transcript abundance of the mitochondrial *ASP1*, cytosolic *ASP2* and chloroplastic *ASP3* genes of aspartate aminotransferase (AspAT) was two- to three-fold higher in the mutant at day and night compared to the WT. By contrast, the transcript level of the asparagine synthetase gene *ASN1* was reduced more than three-fold in the *bou-2* mutant at day compared to the WT and was significantly lower at day compared to night in the mutant (Figure 3). However, the *ASN2* transcript level was enhanced in the mutant at day compared to the WT and compared to night of the mutant. In addition, glutamate dehydrogenase (*GDH1*) was upregulated in the mutant at night compared to the WT and compared to day (Figure 3) together with glutamate decarboxylase (*GAD*) and GABA aminotransferase (*POP2*). Upregulation of these genes indicates changes in Glu production and its use for GABA and succinate synthesis in the mutant (Figure 3). This view is supported by the high amounts of GABA and succinate in the mutant, especially at night (Figure 8A and Table 1).

## Discussion

### Reduced GDC Activity Mediated Alternation of Gene Expression and Metabolite Abundance of the Photorespiratory Pathway

In bou-2, major subunits or isoforms of GDC and SHMT (except SHMT3), the enzymes directly involved in Gly and Ser metabolism, as well as other key enzymes of photorespiration, including GLYK, GOX, HPR, and PGLP were down-regulated at either day, night or both (Figure 2A and Supplementary Figure S2). Despite remaining transcript level of genes encoding GDC subunits, Eisenhut et al. (2013) measured GDC activity as low as 15% of the WT, suggesting its post-transcriptional regulation. As a consequence of reduced GDC activity, glycine cannot be converted to serine and, hence, accumulated to high levels in *bou-2* at both day and night (Figure 1A). In addition to Gly, contents of the photorespiratory intermediates Ser, 2-OG, glycolate and glycerate were higher in the bou-2 mutant (Table 1), suggesting that the flux through photorespiration was partially maintained despite reduced transcript abundances of PR enzymes.

Studies with strong Arabidopsis mutants of PR enzymes revealed elevated substrate (or upstream substrate) and depleted product (or downstream product) pool sizes for the respective enzymes in response to a shift from high to low CO_2_ (Timm et al., 2008, 2012; Eisenhut et al., 2013). In particular, *pglp1* mutants exhibit higher levels of glycolate (likely, 2-p-glycolate; see Eisenhut et al., 2013) and depleted levels of Gly, Ser and glycerate compared to the WT. Levels of Gly are massively enriched in shm1 mutants, while Ser and glycerate are depleted (Eisenhut et al., 2013). In *glyk1* mutants, glycerate as well as Ser, Gly and glycolate are more abundant. The weaker PR mutant hpr1 exhibits higher Ser, Glyc and glycolate levels, although glycerate levels appear unaffected by the mutation.

With reduced GDC activity, the source(s) of highly elevated Ser amounts was (were) of particular interest. Upregulation of major PGDH and PSAT isoforms at night in the bou-2 mutant are in favor of the phosphorylated pathway of Ser synthesis (Figure 2). However, slight down-regulation of PSP1, catalyzing the final step of this pathway, together with down-regulation of the majority of genes encoding enzymes of the CBBC as the source of 3-PGA (Figure 5) contradict this hypothesis. Of the known genes encoding for enzymes catalyzing the glycerate pathway of Ser biosynthesis, only SGAT, which is also involved in photorespiration, was slightly upregulated at day in the bou-2 mutant (Figure 2). The technical inability to distinguish glycerate from 3-PGA with the GC-MS setup applied precludes to draw conclusions from increased levels of glycerate (in particular at day), which hence could support either of the three ways of Ser biosynthesis.

In addition, formate from C1 metabolism can serve as an alternative source for Ser and, therefore, Ser synthesis *via* the C1–THF synthase/SHMT pathway that may constitute an alternative source of Ser used for Cys synthesis (Li et al., 2003). The *Arabidopsis glyD* mutant lacking GDC activity due to a recessive nuclear mutation (Somerville and Ogren, 1982), showed 2.5-fold higher accumulation of Ser in shoots compared to WT after supply of [^13^C] formate (Li et al., 2003). Therefore, the high expression of the plastidial *SHMT3* isoform observed in the *bou–2* mutant in the present study could have contributed to Ser and Cys synthesis *via* C1–THF synthase/SHMT pathway.

### Responses of Sulfate Assimilation to Reduced GDC Activity Can Only Partially Be Explained by Changes in Gene Expression

The results of the present [^35^S]SO_4_^2-^ feeding experiments are consistent with earlier studies with WT *Arabidopsis* showing higher uptake and incorporation of sulfate into GSH, GLS and proteins at day compared to night (Kopriva et al., 1999; Huseby et al., 2013). However, in the present study GSH contents were similar at day and night, while GLS and protein contents were slightly higher at night than at day in WT *Arabidopsis*. Metabolite accumulation does not only depend on its synthesis, but also of its turnover (Huseby et al., 2013). Apparently, sulfur pools are depleted during the day to a higher extent by metabolic reactions than at night, thereby preventing enhanced GSH accumulation at an enhanced flux through the sulfate assimilation pathway during the day. This view is supported by a higher sulfate pool during dark compared to day in the WT. In the present study, the APR gene did not show any changes in its transcript level between day and night (Figure 7). This might be due to differences in the light regime between the present study (12h light) and previous experiments (10 h light or 16 h light) (Kopriva et al., 1999; Huseby et al., 2013). Apparently, also day length affects the transcriptional regulation of APR. Previous studies showed that sulfur assimilation is subject to transcriptional regulation of sulfate transporters and APR by sulfate availability (Takahashi et al., 2011), salt stress (Koprivova et al., 2008), and to transcriptional regulation of mitochondrial SAT (Haas et al., 2008). However, other post-transcriptional and post-translation regulation of sulfur assimilation cannot be excluded. In the present study, plants were pre-cultivated at sufficient sulfur supply that may have suppressed genes of the sulfur assimilatory pathway (Figure 7; Kopriva and Rennenberg, 2004).

In the *bou-2* mutant, uptake of [^35^S]SO_4_^2-^ and its incorporation into GLS was higher at day than at night. RNASeq results of the present study showed significantly higher transcript level of *ATPS2, APK1 APK3* together with *SOT17* and *SOT18* gene isoforms at day in the mutant compared to night. These results are consistent with previous studies of Huseby et al. (2013) indicating that GLS synthesis was regulated at the transcriptional level in *bou-2* mutant. However, the [^35^S]SO_4_^2-^ uptake and fluxes into different pools in *bou-2* mutant were heavily decreased compared to the WT (Figure 6), probably due to the high pool sizes of S metabolites. In addition, the [^35^S]SO_4_^2-^ incorporation into GSH and proteins did not follow day/night regulation (Figure 6). These results indicate a significant disturbance of sulfate primary metabolism in the *bou-2* mutant. SAT and OAS-TL mediate the synthesis of Cys through the formation of the cysteine synthesis complex (CSC) (Wirtz et al., 2012). In the mutant during daylight, mainly the plastidial *SAT1* isoform was responsible for providing O-acetyl-serine (OAS) for Cys synthesis, while OAS was used predominantly by the cytosolic OAS-TL gene for Cys synthesis during day and night compared to WT. However, significant downregulation of *OAS-TL A* in the mutant at day compared to night is inconsistent with a higher Cys content in the mutant during the day. Although other OAS-TL isoforms failed to show any significant changes in transcript levels, a contribution to Cys synthesis cannot be excluded. Apparently, the upregulated *SAT1* and *SAT3* isoforms together with *SiR* provided sufficient provision of substrates for Cys synthesis.

However, sulfate reduction and Cys synthesis can be regulated at post-translation level by protein–protein interaction of the enzymes of this metabolic pathway and/or by sensing levels of downstream products of sulfate assimilation (Koprivova and Kopriva, 2014). The latter is indicated by the observation that external application of Cys as well as GSH decreased the mRNA level and activity of APS reductase (Lappartient et al., 1999; Vauclare et al., 2002). In the present study, feedback inhibition by the high content of Cys and/or GSH in the mutant could be responsible for the low gene expression of *APR1* and, as a consequence, the reduced flux of [^35^S]SO_4_^2-^ in the mutant. Further, the Cys concentration can regulate SATase activity by feed-back inhibition (Noji et al., 1998; Wirtz et al., 2012). *SAT5* is Cys sensitive gene isoform strictly regulating the OAS concentration in the cytosol *via* feed-back inhibition by Cys, while *SAT1* and *SAT3* seem to be Cys insensitive isoforms (Noji et al., 1998; Wirtz et al., 2012). In the present study, the high Cys content of the mutant could have mediated downregulation of Cys sensitive *SAT5* and upregulation of Cys insensitive *SAT1* and *SAT3* (Figure 7).

So far little is known about expression of the gene of ɣ–ECS. Studies of Cobbett et al. (1998) with Arabidopsis *cad2-1* mutant with deletion in the ɣ-ECS gene, reported control of GSH synthesis by transcriptional changes of its key enzyme ɣ–ECS, while studies of May et al. (1998) reported post-translational regulation of ɣ–ECS activity by GSH concentration. Although in present study ɣ–ECS did not show any significant changes in transcript level, slight decrease in transcript level of ɣ–ECS could be mediated by high content of GSH in *bou-2* mutant (Figures 6, 7). In addition, the GSH synthesis is controlled by the availability of its constituent amino acids Cys, Glu, and Gly (Kopriva and Rennenberg, 2004). Poplar leaves supplied with external Cys had enhanced GSH content irrespective of ⁣-ECS and GSHS activity (Noctor et al., 1996). At night, Gly availability can be a limiting factor for GSH synthesis. When poplars are grown at high CO_2_, reduced photorespiratory Gly production leads to a lack of light-induced conversion of ⁣-EC to GSH; under these conditions, feeding poplar leaves with external Gly allowed undisturbed synthesis of GSH during night (Noctor et al., 1998). As both, the Gly and Cys contents are high at light and dark, GSH biosynthesis does not appear to be limited by the availability of these amino acids in the mutant. Apparently, in present study the synthesis of GSH is mediated by availability of its constituent amino acids although ⁣–ECS gene expression is downregulated.

The significantly lower flux of [^35^S]-sulfate into labeled proteins in the mutant at day, and the slightly lower flux at night compared to the WT indicate that Met was not depleted in the mutant by enhanced protein synthesis. The level of S-AdoMet (SAM), synthesized from Met by SAM synthase (SAMS), controls the sulfur flux partitioning between Met and Thr by activating Thr synthase (Kocsis et al., 2003). In present study, low expression of the *SAMS* gene indicates low SAM contents that could not explain upregulation of the *TS* gene in the mutant. However, Met can be synthesized also *via* C1-dependent folate metabolism (Zhang et al., 2010). The low transcript abundance of *MS* and *SAMS* and high transcript level of *SHMT3* at day and night support the view that SHMT controls the C1 flux in favor of Ser over Met production in *bou-2* mutant. Met homeostasis does not depend only on its *de novo* biosynthesis from Asp and Cys in plastids, but also on its regeneration from cytosolic SAM in the S-methylmethionine (SMM) cycle (Kocsis et al., 2003; Ravanel et al., 2004). Thus, Met metabolism is complex and strictly regulated (Hesse et al., 2004; Sauter et al., 2013). Its significance for the consumption of photorespiratory and non-photorespiratory Ser requires further attention.

### Reduced GDC Activity Affects the Crosstalk of Sulfur Assimilation With Nitrogen and Carbon Metabolism

Interaction of C and N metabolism is strongly determined by the operation of the photorespiratory pathway (Nunes-Nesi et al., 2010). In this pathway ammonium is released during conversion of Gly to Ser (Nunes-Nesi et al., 2010 and their references). This photorespiratory N recycling affects N metabolism because it is intimately associated with both respiratory C flow and N assimilation (Novitskaya et al., 2002). Both, the ammonia coming from primary N assimilation and the ammonium released in the photorespiration are assimilated by the GS-GOGAT pathway (Hirel and Lea, 2002). The amount of photorespiratory produced ammonia is up to 10-fold greater than the amount formed by primary N reduction (Keys et al., 1978). Therefore, impaired photorespiration in *bou-2* mutant results in a drastic decrease in ammonia availability and, as a consequence, to decreased transcript levels of GS-GOAGT genes. In the present study, an ammonia deficit is supported by a significantly higher foliar nitrate content of the mutant compared to the WT at both light and dark (data not shown). In the *Arabidopsis ggt1* knock-out mutant of photorespiratory glutamate:glyoxylate aminotransferase 1 (GGT1), nitrogen assimilation was limited due to low carbon assimilation by a decreased RuBisCO content (Dellero et al., 2015). In the *bou-2* mutant similar changes were observed such as downregulation of expression of genes encoding the small subunits of Rubisco (*RBSC*) (Figure 4) together with increased levels of sugars (Table 1; Eisenhut et al., 2013). The same changes were also obtained in experiments with N-deficient *Nicotiana tabacum* plants (Paul and Driscoll, 1997; Nielsen et al., 1998). Therefore, the decrease in *RBSC* transcript level in the present study is thought to be the result of photorespiratory mediated C:N disbalance in the mutant.

N limitation decreases the transcript abundance of genes of amino acid synthesis (Peng et al., 2007) and the amino acid content (Batushansky et al., 2015) but may still result in enhanced levels of Ala, Val, and Ser (Lemaître et al., 2008). In previous study with the *bou-2* mutant, Eisenhut et al. (2013) also observed amino acid accumulation at seedling stage. The present results show increased levels of Ser, Ala, Val, Leu, Ile, Thr at day and, except for Ala and Val, remained high in mutant at night. Since the *bou-2* mutant was not exposed to low N supply, the changes in amino acid metabolism are not consequence of limited nitrogen assimilation *per se* but are caused by impaired photorespiration, leading to altered N metabolism and its cross-talk with C metabolism.

The TCA cycle is a central metabolic hub for the interacting pathways of respiration, N assimilation and photorespiration and an important source of organic carbon intermediates such as 2-OG necessary for synthesis of amino acids (Gauthier et al., 2010). TCA cycle is a major source of 2-OG, however, in illuminated leaves its decarboxylation can be reduced by 80% compared to dark respiration (Foyer et al., 2011 and their references). During the day, 2-OG is thought to be used predominantly for Glu and Gln synthesis, at night predominantly for succinate synthesis (Gauthier et al., 2010). In the present study, low *de novo* synthesis of Glu by the GS/GOGAT pathway could explain accumulation of 2-OG in the mutant during the day. In addition, upregulation of *PK* in the mutant indicates an enhanced supply of pyruvate for reactions of TCA cycle at night. Upregulation of genes upstream of 2-OG and isocitrate depletion indicates the use of these metabolites for 2-OG synthesis. Apparently, glycolysis also provides sufficient amounts of pyruvate for the synthesis of pyruvate derived amino acids (Ala, Val, Ile, Ieu).

When the photosynthetic processes and primary N assimilation are disturbed, it is important for plant survival to still maintain the C:N balance under these conditions. High amounts of 2-OG in the mutant at day may therefore constitute a consequence of intensified transamination between OAA and Glu. Further, Ala can be produced by transamination of pyruvate thereby converting Glu to 2-OG using a glutamate-alanine transaminase (Hildebrandt et al., 2015). By this transamination reaction, the *bou-2* mutant is able to control the interconversion of 2-OG to Glu and the recycling of carbon skeletons and ammonium. In addition, 2-OG can originate from a phosphorylated pathway in which the amino group of Glu is transferred to 3-phosphohydroxypyruvic acid (3-PHP), resulting in one molecule of 2-OG and phosphoserine (PS) each (Benstein et al., 2013). In the *bou-2* mutant, this pathway could be source of 2-OG because of high transcript level of the *PSAT1* gene at day and night compared to the WT. This source of 2-OG will become particularly important at diminished photorespiratory 2-OG synthesis (Benstein et al., 2013).

Glutamate dehydrogenase (GDH) can support aminotransferase action by channeling carbon from Glu into the TCA cycle (Miflin and Habash, 2002). High expression of *GDH* genes and oxidative deamination of Glu to facilitate recycling of C and N was specifically observed at C limitation (Robinson et al., 1991; Aubert et al., 2001). Thus, the GDH shunt could be a useful mechanism to regulate Glu content and to respond to different needs of cells for N and C. In the present study, the transcript level of all three *GDH* isoforms were higher in the mutant at night compared to the WT and to day. Together with upregulation of TCA cycle, lower 2-OG content at night and the downregulation of genes of GS-GOGAT pathway this result supports the assumption that the GDH shunt is engaged in Glu synthesis in the mutant at dark as a consequence of impaired photorespiration. The high amount of GABA found in the *bou-2* mutant may be a consequence of the high Gly level and its inhibitory effect on mitochondrial GABA transaminase (Eisenhut et al., 2013). Studies with *Arabidopsis* mutants lacking several GABA transporters at limited C or N supply indicate the significance of the GABA shunt in regulating N and C partitioning by linking amino acid metabolism and the TCA cycle (Michaeli et al., 2011; Batushansky et al., 2015). In present study, the two-fold higher content of GABA in the mutant at day and night compared to the WT could play a role in maintaining the C:N balance.

The regulatory interaction between sulfate, nitrate and carbon metabolism has been established in number of studies (Reuveny et al., 1980; Koprivova et al., 2000; Wang et al., 2000, 2003; Kopriva et al., 2002; Hesse et al., 2003). Sugars such as sucrose and glucose were shown to regulate S assimilation directly. In *Lemna minor* sucrose restored APR activity and sulfate uptake and the flux through sulfate assimilation that were severely decreased in plants grown in an atmosphere without CO_2_ (Kopriva et al., 2002). Sulfur and nitrogen interact in such a way that the depletion of one reduces the uptake and assimilation of the other. N deficiency in *A. thaliana* lead to reduced activity and transcript level of APR indicating its transcriptional control by N availability, whereas Cys and GSH content were not affected (Koprivova et al., 2000). Thus, photorespiration affects sulfur metabolism indirectly by its cross-talk with C and N metabolism. In the *bou-2* mutant, impaired photorespiration reduces photosynthesis, mediates C:N disbalance, and leads to decreased N assimilation, thereby reducing the *APR* transcript level and sulfate flux. Under these conditions, still Cys and GSH accumulated, presumably due to lower consumption in anabolic and/or catabolic processes. This alludes that changes in sulfate metabolism in the *bou-2* mutant are not a consequences of N deficiency *per se* yet of complex interactions and crosstalk of S, C and N metabolism at impaired photorespiration.

## Conclusion

In the present study [^35^S]SO_4_^2-^ flux experiment showed that Ser produced by photorespiration is the dominant precursor of Cys synthesis in photosynthetic tissue of *A. thaliana* WT during light (hypothesis 1). In addition, results of fluxes through the sulfur assimilation pathway together with content of sulfate containing metabolites showed that photorespiratory Ser biosynthesis in WT plants cannot be replaced by phosphorylated Ser production during night (contradicting our hypothesis 2). However, these changes were not followed by day/night changes in transcript level of genes of sulfate reduction and assimilation pathway presumably due to growing preconditions (contradicting our hypothesis 3). Impaired photorespiration significantly disturbed sulfate metabolism in the *bou-2* mutant. The [^35^S]SO_4_^2-^ uptake and fluxes in mutant were heavily decreased while the content of sulfur-rich metabolites were strongly increased compared to the WT. The results showed that high amount of Cys and other sulfur-rich metabolites do not originate from alternative phosphorylated Ser production (contradicting our hypothesis 2). However, C1–THF synthase/SHMT pathway could contribute to Ser and therefore Cys synthesis in *bou-2* mutant and should be further investigated. The RNAseq results showed that changes in sulfate reduction and assimilation in *bou-2* mutant could only be partially explained by transcriptional regulation (hypothesis 3). Besides the changes in sulfate metabolism, the reduced GDC activity in *bou-2* mutant deregulates CBBC and TCA, cycle, glycolysis, amino acid synthesis, and nitrogen and carbon assimilation. Thus, changes in sulfate metabolism in *bou-2* mutant are consequences of complex interaction and crosstalk of S, C and N metabolism. The photorespiratory Ser production can be replaced by other metabolic Ser sources but this has as a consequence the disruption of cross-talk of S, C and N metabolism. For a better understanding of the regulation of S assimilation and its crosstalk with C and N metabolism at impaired photorespiration, further studies of transcription factors and specific sensing mechanisms are required.

## Data Availability

The read data have been submitted to the National Center for Biotechnology Information Gene Expression Omnibus under accession number GSE86380 (https://www.ncbi.nlm.nih.gov/geo/query/acc.cgi?acc=GSE86380).

## Author Contributions

HR and AW conceived the experiments. SS and NR designed the experiments. SS, NR, and SF performed the experiments. SS, DB, and TM-A analyzed data. SS and DB generated the figures and tables. SK, TM-A, LA, NR, and FK contributed reagents, materials, and analytical tools. SS, DB, HR, and SA wrote the manuscript.

## Conflict of Interest Statement

The authors declare that the research was conducted in the absence of any commercial or financial relationships that could be construed as a potential conflict of interest.

## Footnotes

1 http://www.R-project.org

2 http://www.bioinformatics.babraham.ac.uk/projects/fastqc/

3 http://www.arabidopsis.org/

## References

[B1] Amir R. Hacham Y. Galili G. (2002). Cystatione γ-synthase and threonine synthase operate in concert to regulate carbon flow towards methionine in plants. *Trends Plant Sci.* 7 153–156. 10.1016/S1360-1385(02)02227-6 11950610

[B2] Aubert S. Bligny R. Douce R. Gout E. Ratcliffe R. G. Roberts J. K. M. (2001). Contribution of glutamate dehydrogenase to mitochondrial glutamate metabolism studied by 13C and 31P nuclear magnetic resonance. *J. Exp. Bot.* 52 37–45. 10.1093/jexbot/52.354.37 11181711

[B3] Batushansky A. Kirma M. Grillich N. Pham P. A. Rentsch D. Galili G. (2015). The transporter GAT1 plays an important role in GABA-mediated carbon-nitrogen interactions in Arabidopsis. *Front. Plant Sci.* 6 1–10. 10.3389/fpls.2015.00785 26483804 PMC4586413

[B4] Benjamini Y. Hochberg Y. (1995). Controlling the false discovery rate: a practical and powerful approach to multiple testing. *J. R. Stat. Soc.* 57 289–300.

[B5] Benstein R. M. Ludewig K. Wulfert S. Wittek S. Gigolashvili T. Henning Frerigmann H. (2013). Arabidopsis phosphoglycerate dehydrogenase1 of the phosphoserine pathway is essential for development and required for ammonium assimilation and tryptophan biosynthesis. *Plant Cell* 25 5011–5029. 10.1105/tpc.113.118992 24368794 PMC3904002

[B6] Brader G. Mikkelsen M. D. Halkier B. A. Palvia E. T. (2006). Altering glucosinolate profiles modulates disease resistance in plants. *Plant J.* 46 758–767. 10.1111/j.1365-313X.2006.02743.x 16709192

[B7] Bradford M. M. (1976). A rapid and sensitive method for the quantification of microgram quantities of protein utilizing the principle of protein-dye binding. *Anal. Biochem.* 72 248–254. 10.1016/0003-2697(76)90527-3 942051

[B8] Bray N. L. Pimentel H. Melsted P. Pachter L. (2016). Near-optimal probabilistic RNA-seq quantification. *Nat. Biotechnol.* 34 525–527. 10.1038/nbt.3519 27043002

[B9] Brown P. D. Tokuhisa J. G. Reichelt M. Gershenzon J. (2003). Variation of glucosinolates accumulation among different organs and developmental stages of *Arabidopsis thaliana*. *Phytochemistry* 62 471–481. 10.1016/S0031-9422(02)00549-6 12620360

[B10] Cobbett C. S. May M. J. Howden R. Rolls B. (1998). The glutathione-deficient, cadmium-sensitive mutant, cad2–1, of *Arabidopsis thaliana* is deficient in γ-glutamylcysteine synthetase. *Plant J.* 16 73–78. 10.1046/j.1365-313x.1998.00262.x 9807829

[B11] Dellero Y. Lamothe-Sibold M. Jossier M. Hodges M. (2015). *Arabidopsis thaliana* ggt1 photorespiratory mutants maintain leaf carbon/nitrogen balance by reducing RuBisCO content and plant growth. *Plant J.* 83 1005–1018. 10.1111/tpj.12945 26216646

[B12] Eisenhut M. Planchais S. Cabassa C. Guivarc´h A. Justin A.-M. Taconnat L. Renou J.-P. (2013). Arabidopsis a bout de souffle is a putative mitochondrial transporter involved in photorespiratory metabolism and is required for meristem growth at ambient CO2 levels. *Plant J.* 73 836–849. 10.1111/tpj.12082 23181524

[B13] Eisenhut M. Hocken N. Weber A. P. (2015). Plastidial metabolite transporters integrate photorespiration with carbon, nitrogen, and sulfur metabolism. *Cell Calcium* 58 98–104. 10.1016/j.ceca.2014.10.007 25465893

[B14] Engel N. Ewald R. Gupta K. J. Zrenner R. Hagemann M. Bauwe H. (2011). The presequence of *Arabidopsis* serine hydroxymethyltransferase SHM2 selectively prevents import into mesophyll mitochondria. *Plant Physiol.* 157:1711–1720. 10.1104/pp.111.184564 21976482 PMC3327202

[B15] Fiehn O. Kopka J. Dörmann P. Altmann T. Trethewey R. N. Willmitzer L. (2000). Metabolite profiling for plant functional genomics. *Nat. Biotechnol.* 18 1157–1161. 10.1038/81137 11062433

[B16] Foyer C. H. Noctor G. (2000). Oxygen processing in photosynthesis: regulation and signalling. *Rev. Lit. Arts Am.* 146 359–388. 10.1093/jxb/erv018 25740923 PMC4378640

[B17] Foyer C. H. Noctor G. Hodges M. (2011). Respiration and nitrogen assimilation: targeting mitochondria-associated metabolism as a means to enhance nitrogen use efficiency. *J. Exp. Bot.* 62 1467–1482. 10.1093/jxb/erq453 21282329

[B18] Gauthier P.P.G. Bligny R. Gout E. Mahé A. Nogués S. Hodges M. Tcherkez G.G.B. (2010). In folio isotopic tracing demonstrates that nitrogen assimilation into glutamate is mostly independent from current CO2 assimilation in illuminated leaves of *Brassica napus*. *New Phytol.* 185 988–999. 10.1111/j.1469-8137.2009.03130.x 20070539

[B19] Gullner G. Kömives T. Rennenberg H. (2001). Enhanced tolerance of transgenic poplar plants overexpressing gamma-glutamylcysteine synthetase towards chloroacetanilide herbicides. *J. Exp. Bot.* 52 971–979. 10.1093/jexbot/52.358.971 11432914

[B20] Gupta D. K. Huang H. G. Yang X. E. Razafindrabe B. H. N. Inouhe M. (2010). The detoxification of lead in *Sedum alfredii* H. is not related to phytochelatins but the glutathione. *J. Hazard. Mater.* 177 437–444. 10.1016/j.jhazmat.2009.12.052 20047791

[B21] Haas F. H. Heeg C. Queiroz R. Bauer A. Wirtz M. Hell R. (2008). Mitochondrial serine acetyltransferase functions as a pacemaker of cysteine synthesis in plant cells. *Plant Physiol.* 148 1055–1067. 10.1104/pp.108.125237 18753283 PMC2556817

[B22] He J. Li H. Ma C. Zhang Y. Polle A. Rennenberg H. (2015). Overexpression of bacterial γ-glutamylcysteine synthetase mediates changes in cadmium influx, allocation and detoxification in poplar. *New Phytol.* 205 240–254. 10.1111/nph.13013 25229726

[B23] Herschbach C. Gessler A. Rennenberg H. (2012). Long-distance transport and plant internal cycling of N- and S-compounds. *Progr. Bot.* 73 161–188. 10.1007/978-3-642-22746-2_6

[B24] Herschbach C. van der Zalm E. Schneider A. Jouanin L. De Kok L. J. Rennenberg H. (2000). Regulation of sulphur nutrition in wild type and transgenic poplar over-expresing γ-glutamylsysteine synthetase in the cytosol as affected by atmospheric H2S. *Plant Physiol.* 124 461–473. 10.1104/pp.124.1.461 10982459 PMC59159

[B25] Hesse H. Kreft O. Maimann S. Zeh M. Hoefgen R. (2004). Current understanding of the regulation of methionine biosynthesis in plants. *J. Exp. Bot.* 55 1799–1808. 10.1093/jxb/erh139 15234989

[B26] Hesse H. Trachsel N. Suter M. Kopriva S. von Ballmoos P. Rennenberg H. (2003). Effect of glucose on assimilatory sulfate reduction in roots of *Arabidopsis thaliana*. *J. Exp. Bot.* 54 1701–1709. 10.1093/jxb/erg177 12754263

[B27] Hildebrandt T. M. Nesi A. N. Araújo W. L. Braun H. P. (2015). Amino acid catabolism in plants. *Mol. Plant.* 8 1563–1579. 10.1016/j.molp.2015.09.005 26384576

[B28] Hirel B. Lea P.J. (2002). “The biochemistry, molecular biology and genetic manipulation of primary ammonia assimilation,” in *Photosynthetic Nitrogen Assimilation and Associated Carbon and Respiratory Metabolism. Advances in Photosynthesis and Respiration* vol. 12 eds Foyer C. H. Noctor G. (Amsterdam: Springer).

[B29] Huseby S. Koprivova A. Lee B. R. Saha S. Mithen R. Wold A.-B. (2013). Diurnial and light regulation of sulphur assimilation and glucosinolate biosynthesis in Arabidopsis. *J. Exp. Bot.* 64 1039–1048. 10.1093/jxb/ers378 23314821 PMC3580815

[B30] Jang J. C. León P. Zhou L. Sheen J. (1997). Hexokinase as a sugar sensor in higher plants. *Plant Cell* 9 5–19. 10.1105/tpc.9.1.5 9014361 PMC156897

[B31] Keys A. J. Bird I. F. Cornelius M. J. Lea P. J. Miflin B. J. Wallsgroove R. M. (1978). Photorespiratory nitrogen cycle. *Nature* 275 741–743. 10.1038/275741a0

[B32] Kocsis M. G. Ranocha P. Gage D. A. Simon E. S. Rhodes D. Peel G. J. (2003). Insertional inactivation of the methionine S-Methyltransferase gene eliminates the S-Methylmethionine cycle and increases the methylation ratio. *Plant Physiol.* 131 1808–1815. 10.1104/pp.102.018846 12692340 PMC166937

[B33] Kopriva S. Muheim R. Koprivova A. Trachsel N. Catalano C. Suter M. (1999). Light regulation of assimilatory sulfate reduction in *Arabidopsis thaliana*. *Plant J.* 20 37–44 10.1046/j.1365-313X.1999.00573.x 10571863

[B34] Kopriva S. Rennenberg H. (2004). Control of sulphate assimilation and glutathione synthesis: interaction with N and C metabolism. *J. Exp. Bot.* 55 1831–1842. 10.1093/jxb/erh203 15286142

[B35] Kopriva S. Suter M. von Ballmoos P. Hesse H. Krähenbühl U. Rennenberg H. (2002). Interaction of sulfate assimilation with carbon and nitrogen metabolism in *Lemna minor*. *Plant Physiol.* 122 737–746. 10.1104/pp.007773 12428005 PMC166659

[B36] Koprivova A. Kopriva S. (2014). Molecular mechanisms of regulation of sulfate assimilation: first steps on long road. *Front. Plant Sci.* 5 1–10. 10.3389/fpls.2014.00589 25400653 PMC4212615

[B37] Koprivova A. North K. A. Kopriva S. (2008). Complex signaling network in regulation of adenosine 5′-phosphosulfate reductase by salt stress in *Arabidopsis* roots. *Plant Physiol.* 146 1408–1420. 10.1104/pp.107.113175 18218969 PMC2259037

[B38] Koprivova A. Suter M. den Camp R. O. Brunold C. Kopriva S. (2000). Regulation of sulfate assimilation by nitrogen in arabidopsis. *Plant Physiol.* 122 737–746. 10.1104/pp.122.3.737 10712537 PMC58909

[B39] Lappartient A. G. Vidmar J. J. Leustek T. Glass A. D. M. Touriane B. (1999). Inter-organ signalling in plants: regulation of ATP sulfurylase and sulfate transporter genes expression in roots mediated by phloem-translocated compound. *Plant J.* 18 89–95. 10.1046/j.1365-313X.1999.00416.x 10341446

[B40] Lemaître T. Laure Gaufichon L. Boutet-Mercey S. Christ A. Masclaux-Daubresse C. (2008). Enzymatic and metabolic diagnostic of nitrogen deficiency in *Arabidopsis thaliana* wassileskija accession. *Plant Cell Physiol.* 49 1056–1065. 10.1093/pcp/pcn081 18508804

[B41] Li R. Moore M. King J. (2003). Investigating the regulation of one-carbon metabolism in *Arabidopsis thaliana*. *Plant Cell Physiol.* 44 233–241. 10.1093/pcp/pcg029 12668769

[B42] May M. J. Vernoux T. Sanchez-Fernandez R. Van Montagu M. Inzé D. (1998). Evidence for posttranscriptional activation of gamma-glutamylcysteine synthetase during plant stress responses. *Proc. Natl. Acad. Sci. U.S.A.* 95 12049–12054. 10.1073/pnas.95.20.12049 9751788 PMC21763

[B43] Michaeli S. Fait A. Lagor K. Nunes-Nesi A. Grillich N. Yellin A. (2011). A mitochondrial GABA permease connects the GABA shunt and the TCA cycle, and is essential for normal carbon metabolism. *Plant J.* 67 485–498. 10.1111/j.1365-313X.2011.04612.x 21501262

[B44] Miflin B. J. Habash D. Z. (2002). The role of glutamine synthetase and glutamate dehydrogenase in nitrogen assimilation and possibilities for improvement in the nitrogen utilization of crops. *J. Exp. Bot.* 53 979–987. 10.1093/jexbot/53.370.979 11912240

[B45] Mugford S. G. Lee B-R. Koprivova A. Matthewman C. Kopriva S. (2011). Control of sulfur portioning between primary and secondary metabolism. *Plant J.* 65 96–105. 10.1111/j.1365-313X.2010.04410.x 21175893

[B46] Murashige T. Skoog F. (1962). A revised medium for rapid growth and bioassays with tobacco tissue cultures. *Physiol. Plant* 15 473–497. 10.1111/j.1399-3054.1962.tb08052.x

[B47] Nielsen T. H. Krapp A. Röper-Schwarz K. U. Stitt M. (1998). The sugar-mediated regulation of genes encoding the small subunit of Rubisco and the regulatory subunit of ADP glucose pyrophosphorylase is modified by phosphate and nitrogen. *Plant Cell Environ.* 21 443–454. 10.1046/j.1365-3040.1998.00295.x

[B48] Noctor G. Arisi A. C. M. Jouanin L. Kunert K. J. Rennenberg H. Foyer C. H. (1998). Glutathione: biosynthesis, metabolism and relationship to stress tolerance explored in transformed plants. *J. Exp. Bot.* 49 623–647. 10.1093/jxb/49.321.623

[B49] Noctor G. Mhamdi A. Chaouch S. Han Y. Neukermans J. Marquez-Garcia B. (2012). Glutathione in plants: an integrated overview. *Plant Cell Environ.* 35 454–484. 10.1111/j.1365-3040.2011.02400.x 21777251

[B50] Noctor G. Strohm M. Jouanin L. Kunert K. J. Foyer C. H. Rennenberg H. (1996). Synthesis of glutathione in leaves of transgenic poplar overexpressing [gamma]-glutamylcysteine synthetase. *Plant Physiol.* 112 1071–1078. 10.1104/pp.112.3.1071 12226433 PMC158033

[B51] Noji M. Inoue K. Kimura N. Gouda A. Saito K. (1998). Isoform-dependent differences in feedback regulation and subcellular localization of serine acetyltransferase involved in cysteine biosynthesis from *Arabidopsis thaliana*. *J. Biol. Chem.* 273 32739–32745. 10.1074/jbc.273.49.32739 9830017

[B52] Novitskaya L. Trevanion S. J. Driscoll S. Foyer C. H. Noctor G. (2002). How does photorespiration modulate leaf amino acid contents? A dual approach through modelling and metabolite analysis. *Plant Cell Environ.* 25 821–835. 10.1046/j.1365-3040.2002.00866.x

[B53] Nunes-Nesi A. Fernie A. R. Stitt M. (2010). Metabolic and signaling aspects underpinning the regulation of plant carbon nitrogen interactions. *Mol. Plant* 3 973–996. 10.1093/mp/ssq049 20926550

[B54] Paul M. J. Driscoll S. P. (1997). Sugar repression of photosynthesis: the role of carbohydrates in signalling nitrogen deficiency through source: sink imbalance. *Plant Cell Environ.* 9 783–798. 10.1046/j.1365-3040.1997.d01-17.x

[B55] Peng M. Bi Y-M. Zhu T. Rothstein S. J. (2007). Genome-wide analysis of *Arabidopsis* responsive transcriptome to nitrogen limitation and its regulation by the ubiquitin ligase gene NLA. *Plant Mol. Biol.* 65 775–797. 10.1007/s11103-007-9241-0 17885809

[B56] Pimentel H. J. Bray N. Puente S. Melsted P. Pachter L. (2016). Differential analysis of RNA-Seq incorporating quantification uncertainty. *Nat. Methods* 14 687–690. 10.1038/nmeth.4324 28581496

[B57] Piotrowski M. Schemenwitz A. Lopukhina A. Müller A. Janowitz T. Weiler E. W. (2004). Desulfoglucosinolate sulfotransferases from *Arabidopsis thaliana* catalyze the final step in the biosynthesis of the glucosinolate core structure. *J. Biol. Chem.* 279 50717–50725. 10.1074/jbc.M407681200 15358770

[B58] Plaxton W. C. (1996). The organization and regulation of plant glycolysis. *Annu. Rev. Plant Biol.* 47 (1): 185–214. 10.1146/annurev.arplant.47.1.185 15012287

[B59] Rademacher N. Kern R. Fujiwara T. Mettler-Altmann T. Miyagishima S.-Y. Hagemann M. (2016). Photorespiratory glycolate oxidase is essential for the survival of the red alga *Cyanidioschyzon merolae* under ambient CO2 conditions. *J. Exp. Bot.* 67 3165–3175. 10.1093/jxb/erw118 26994474 PMC4867895

[B60] Ravanel S. Block M. A. Rippert P. Jabrin S. Curien G. Rébeillé F. (2004). Methionine metabolism in plants. *J. Biol. Chem.* 279 22548–22557. 10.1074/jbc.M313250200 15024005

[B61] Rennenberg H. Herschbach C. (2014). A detailed view on sulphur metabolism at the cellular and whole-plant level illustrates challenges in metabolite flux analyses. *J. Exp. Bot.* 65 5711–5724. 10.1093/jxb/eru315 25124317

[B62] Reuveny Z. Dougall D. K. Trinity P. M. (1980). Regulatory coupling of nitrate and sulfate assimilation pathways in cultured tobacco cells. *Proc. Natl. Acad. Sci. U.S.A.* 77 6670–6672. 10.1073/pnas.77.11.6670 16592917 PMC350349

[B63] Robinson S. A. Slade A. P. Fox G. G. Phillips George Ratcliffe R. Stewart G. R. (1991). The role of glutamate dehydrogenase in plant nitrogen metabolism. *Plant Physiol.* 95 509–516. 10.1104/pp.95.2.509 16668014 PMC1077561

[B64] Ros R. Cascales-Miñana B. Segura J. Anoman A. D. Toujani W. Flores-Tornero M. Rosa-Tellez S. Muñoz-Bertomeu J. (2013). Serine biosynthesis by photorespiratory and non-photorespiratory pathways: an interesting interplay with unknown regulatory networks. *Plant Biol.* 15 707–712. 10.1111/j.1438-8677.2012.00682.x 23199004

[B65] Rosso M. G. Yong L. Strizhov N. Reiss B. Dekker K. Weisshaar B. (2003). An *Arabidopsis thaliana* T-DNA mutagenized population (GABI-Kat) for flanking sequence tag-based reverse genetics. *Plant Mol. Biol.* 53 247–259. 10.1023/B:PLAN.0000009297.37235.4a 14756321

[B66] Sauter M. Moffatt B. Saechao M. C. Hell R. Wirtz M. (2013). Methionine salvage and S-adenosylmethionine: essential links between sulfur, ethylene and polyamine biosynthesis. *Biochem. J.* 451 145–154. 10.1042/BJ20121744 23535167

[B67] Scheerer U. Haensch R. Mendel R. R. Kopriva S. Rennenberg H. Herschbach C. (2010). Sulphur flux through the sulphate assimilation pathway is differently controlled by adenosine 5′ phosphosulphate reductase under stress and in transgenic poplar plants overexpressing γ-ECS, SO, or APR. *J. Exp. Bot.* 61 609–622. 10.1093/jxb/erp327 19923196 PMC2803220

[B68] Schupp R. Rennenberg H. (1988). Diurnal changes in the glutathione content of spruce needles (*Picea abies* L.). *Plant Sci.* 57 113–117. 10.1016/0168-9452(88)90076-3

[B69] Somerville C. R. Ogren W. L. (1982). Mutants of the cruciferous plant *Arabidopsis thaliana* lacking glycine decarboxylase activity. *Biochem. J.* 202 373–380. 10.1042/bj2020373 6807291 PMC1158121

[B70] Strohm M. Jouanin L. Kunert K. J. Pruvost C. Polle A. Foyer C. H. (1995). Regulation of glutathione synthesis in leaves of transgenic poplar (*Populus tremula* x P. alba) overexpressing glutathione synthetase. *Plant J.* 7 141–145. 10.1046/j.1365-313X.1995.07010141.x

[B71] Takahashi H. Kopriva S. Giordano M. Saito K. Hell R. (2011). Sulfur assimilation in photosynthetic organisms: molecular function and regulation of transporters and assimilatory enzymes. *Annu. Rev. Plant Biol.* 62 157–184. 10.1146/annurev-arplant-042110-103921 21370978

[B72] Timm S. Mielewczik M. Florian A. Frankenbach S. Dreissen A. Hocken N. (2012). High-to-low CO2 acclimation reveals plasticity of the photorespiratory pathway and indicates regulatory links to cellular metabolism of *Arabidopsis*. *PLoS One* 7:e42809. 10.1371/journal.pone.0042809 22912743 PMC3422345

[B73] Timm S. Nunes-Nesi A. Pärnik T. Morgenthal K. Wienkoop S. Keerberg O. (2008). A cytosolic pathway for the conversion of hydroxypyruvate to glycerate during photorespiration in Arabidopsis. *Plant Cell* 20 2848–2859. 10.1105/tpc.108.062265 18952776 PMC2590732

[B74] Vauclare P. Kopriva S. Fell D. Suter M. Sticher L. Von Ballmoos P. (2002). Flux control of sulfate assimilation in *Arabidopsis thaliana*: adenosine 5′-phosphosulphate reductase is more susceptible than ATP sulphurylase to negative control by thiols. *Plant J.* 31 729–740. 10.1046/j.1365-313X.2002.01391.x 12220264

[B75] Voll L. M. Jamai A. Renné P. Voll H. McClung R. Weber A. P. M. (2006). The photorespiratory arabidopsis shm1 mutant is deficient in SHM1. *Plant Physiol.* 140 59–66. 10.1104/pp.105.071399 16339799 PMC1326031

[B76] Wang R. Guegler K. LaBrie S. T. Crawford N. M. (2000). Genomic analysis of a nutrient response in *Arabidopsis* reveals diverse expression patterns and novel metabolic and potential regulatory genes induced by nitrate. *Plant Cell* 12 1491–1509. 10.1105/tpc.12.8.1491 10948265 PMC149118

[B77] Wang R. Okamoto M. Xing X. Crawford N. M. (2003). Microarray analysis of the nitrate response in *Arabidopsis* roots and shoots reveals over 1,000 rapidly responding genes and new linkages to glucose, trehalose-6-phosphate, iron, and sulfate metabolism. *Plant Physiol.* 132 556–567. 10.1104/pp.103.021253 12805587 PMC166997

[B78] Wirtz M. Beard K. F. M. Lee C. P. Boltz A. Schwarzländer M. Fuchs C. (2012). Mitochondrial cysteine synthase complex regulates O-Acetylserine biosynthesis in plants. *J. Biol. Chem.* 287 27941–27947. 10.1074/jbc.M112.372656 22730323 PMC3431704

[B79] Zhang Y. Sun K. Sandoval F. J. Santiago K. Roje S. (2010). One-carbon metabolism in plants: characterization of a plastid serine hydroxymethyltransferase. *Biochem. J.* 430 97–105. 10.1042/BJ20100566 20518745

