# Supplementary material for: The Photorespiratory BOU Gene Mutation Alters Sulfur Assimilation and Its Crosstalk With Carbon and Nitrogen Metabolism in Arabidopsis thaliana
Source: Front Plant Sci. 2018 Nov 27;9:1709. doi: 10.3389/fpls.2018.01709 (PMC6284229; doi:10.3389/fpls.2018.01709)
Supplement: Supplementary file 2 [file Data_Sheet_2.PDF]

Supplemental Method S1

Confirmation of genuine single mutant *bou-2*

Although the T-DNA insertion line utilized in this study is well established and had been used in a number of previous studies, we wanted to make use of the extensive RNASeq data yielded here to check for possible second site T-DNA insertions. The line was produced with the T-DNA vector pAC161 (details see https://www.gabi-kat.de/db/showseq.php?term2=079D12).
All Illumina paired-end fastq files were aligned against the T-DNA insertion sequence (from left to right border; retrieved from NCBI, https://www.ncbi.nlm.nih.gov/nuccore/AJ537514?report=GenBank) using BWA-MEM (version bwa-0.7.1, available at http://bio-bwa.sourceforge.net) with options -w 2 (keeping only alignments with gaps no longer than 2 bases), -k 50 (keeping only alignments with a minimum length of 50) and -L 0,0 (preventing any soft clipping at the 5' and 3' ends in order to capture alignments that border the insertion, but include non-matching parts, i.e. resulting from the part of the gene where the insertion occurred). The T-DNA border aligned reads were then mapped against the TAIR10 genome (TAIR10_Chr.all.fasta retrieved from https://www.araport.org) using BWA-MEM in default mode. Quantification using htseq-count in default mode (HTSeq version 0.10.0, Anders et al., 2015) including the genome annotation Araport11_GFF3_genes_transposons.201606.gtf (retrieved from https://www.araport.org) confirmed a significant mapping count (read counts >110 per library) only for the locus At5g46800 (encoding BOU), while other genes were randomly hit with background coverage (read counts <9).

Reference

Simon Anders, Paul Theodor Pyl, Wolfgang Huber; HTSeq—a Python framework to work with high-throughput sequencing data, *Bioinformatics*, Volume 31, Issue 2, 15 January 2015, Pages 166–169, https://doi.org/10.1093/bioinformatics/btu638
