# Supplementary material for: The Photorespiratory BOU Gene Mutation Alters Sulfur Assimilation and Its Crosstalk With Carbon and Nitrogen Metabolism in Arabidopsis thaliana
Source: Front Plant Sci. 2018 Nov 27;9:1709. doi: 10.3389/fpls.2018.01709 (PMC6284229; doi:10.3389/fpls.2018.01709)
Supplement: Supplementary file 3 [file Image_1.pdf]

Reduced GDC activity and day/night affect general transcript and metabolite abundances

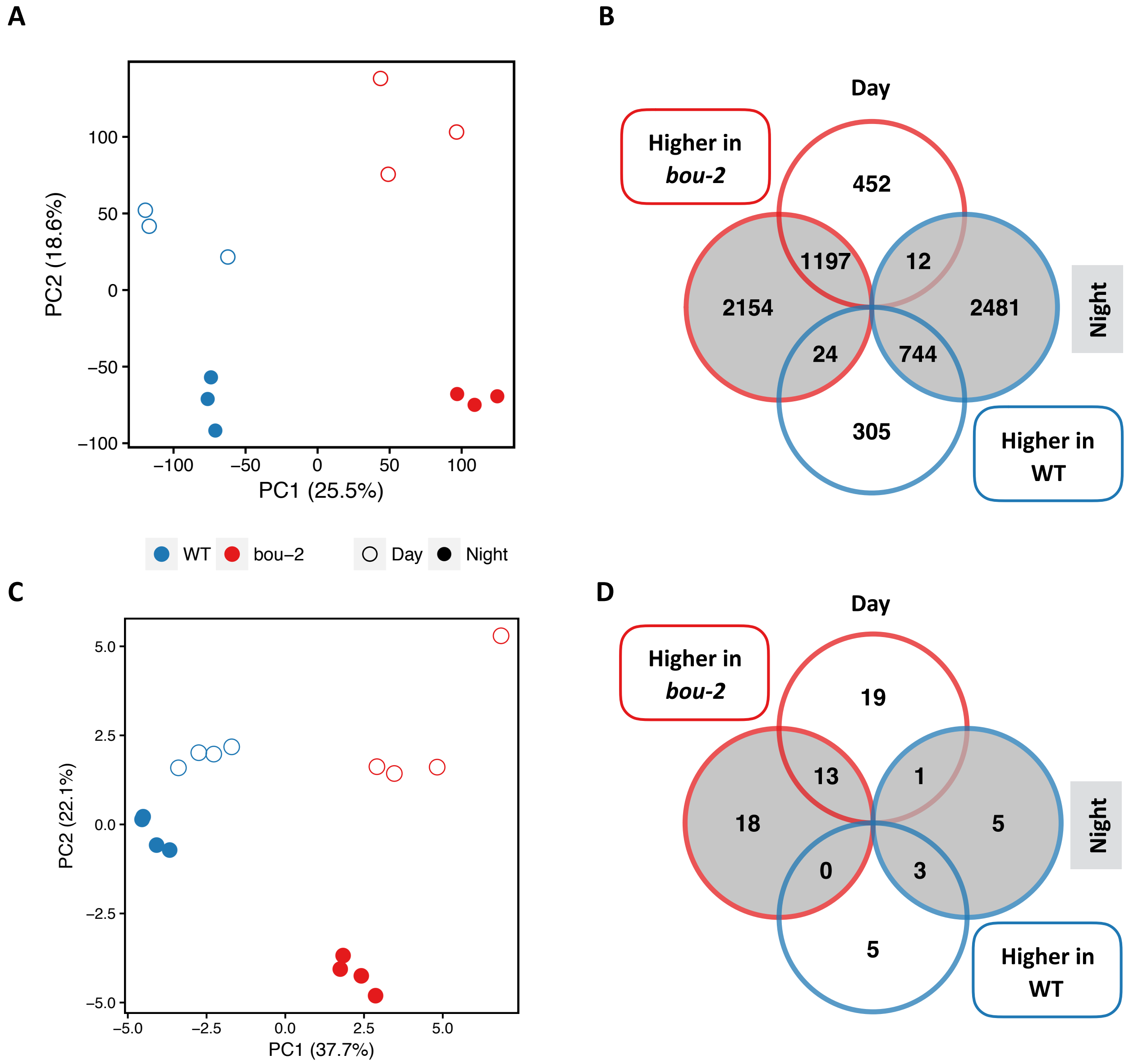

Supplemental Fig S1. Differences between the leaf transcriptomes (A and B) and metabolomes (C and D) of WT (blue) and *bou-2* (red) plants. A and C, Principal component analysis (components 1 and 2) of transcript abundances (A) and relative metabolite levels (C) within the three biological replicates. Open circles, day; filled circles, night. B, Venn diagram of genes identified as differentially expressed between WT and *bou-2* at the respective time points (Wald test implemented in sleuth, FDR < 0.01). For details see Supplemental Dataset S1. D, Venn diagram of metabolites identified as differentially abundant between WT and *bou-2* at the respective time points (Student's T-Test, $p < 0.05$).

To assess the global consequences of reduced GDC activity the bou-2 and WT plants were subjected to RNASeq analysis during the day and the night. Statistical analysis using Wald test implemented in sleuth (Pimentel et al., 2016) revealed a clear discrimination of foliar transcripts between the genotypes and times of day with a total of 7,369 differentially expressed transcripts (DEG) at a false-discovery rate of 0.01% (FDR < 0.01, Suppl. Fig S2 A,B, Supplemental Dataset S1). The number of DEGs was higher at night (6,612 DEGs) compared to day (2,734 DEGs). While 1,197 transcripts were more abundant in bou-2 compared to the wild type (WT) at both times, 2,154 and 452 transcripts were exclusively more abundant at night or day, respectively (Suppl. Fig. S2B). Higher abundance in the WT was detected for 744 transcripts at both times, while 2,481 and 305 transcripts were exclusively more abundant at night or day, respectively.

Principal component analysis (PCA) and venn diagrams were used to compare foliar metabolite abundances between genotypes and times of day. PCA showed a clear discrimination of metabolites between genotypes and times of day (Suppl. Fig. S2C). Venn diagrams identified 64 differentially abundant metabolites between WT and the mutant at day and night (Suppl. Fig. S2D). In general, the metabolites analyzed were more abundant in the mutant than in WT plants. In the mutant, a similar number of metabolites had exclusively higher contents at day or night, while thirteen metabolites were equally abundant at both times (Suppl. Fig. S2D).

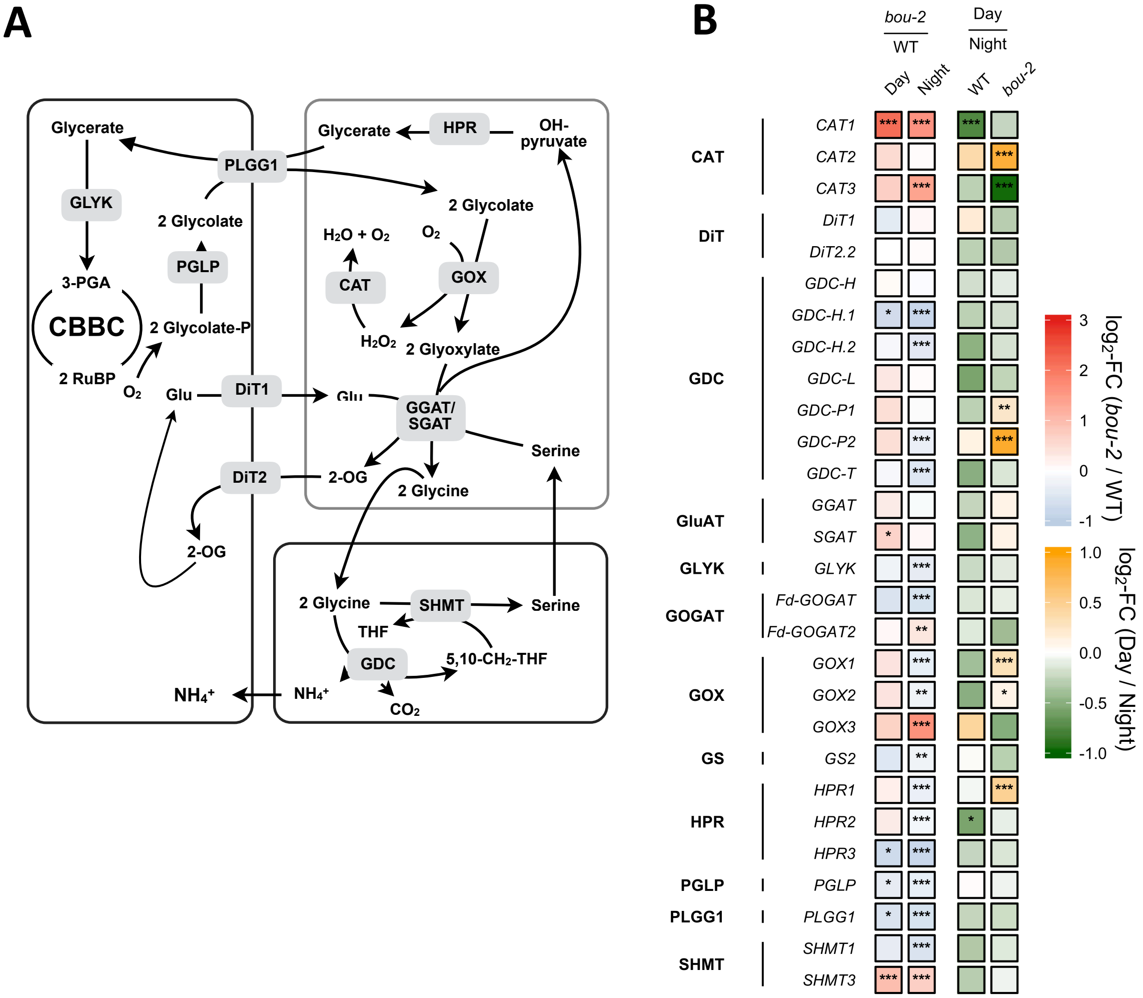

Supplemental Fig S2. A, Schematic presentation of photorespiration. B, Relative levels of genes encoding for enzymes and transporters highlighted in grey boxes in A. Left two tiles of each metabolite or gene represent the $\log_2$-fold change (FC) between *bou-2* and WT under both day (D) and night (N) treatment with false color code ranging from red (higher in *bou-2*) to blue (higher in WT). Right two tiles represent the $\log_2$-fold change (FC) between day and night within WT and *bou-2* with false color code ranging from green (higher at night) to yellow (higher at day). Asterisks indicate differential gene expression as determined by Sleuth (* FDR < 0.05, ** FDR < 0.01, and *** FDR < 0.001). 2-OG, 2-oxoglutarate; Glu, glutamate; THF, tetrahydrofolate; CAT, catalase; DiT, dicarboxylate transporter; GDC, glycine decarboxylase; GGAT, glutamate:glyoxylate aminotransferase; GLYK, glycerate kinase; GOX, Glycolate oxidase; HPR, peroxisomal hydroxypyruvate reductase; PGLP, Phosphoglycolate phosphatase; PLGG, plastidic glycolate glycerate transporter; SGAT, Serine-glutamate aminotransferase; SHMT, serine transhydroxymethyltransferase.

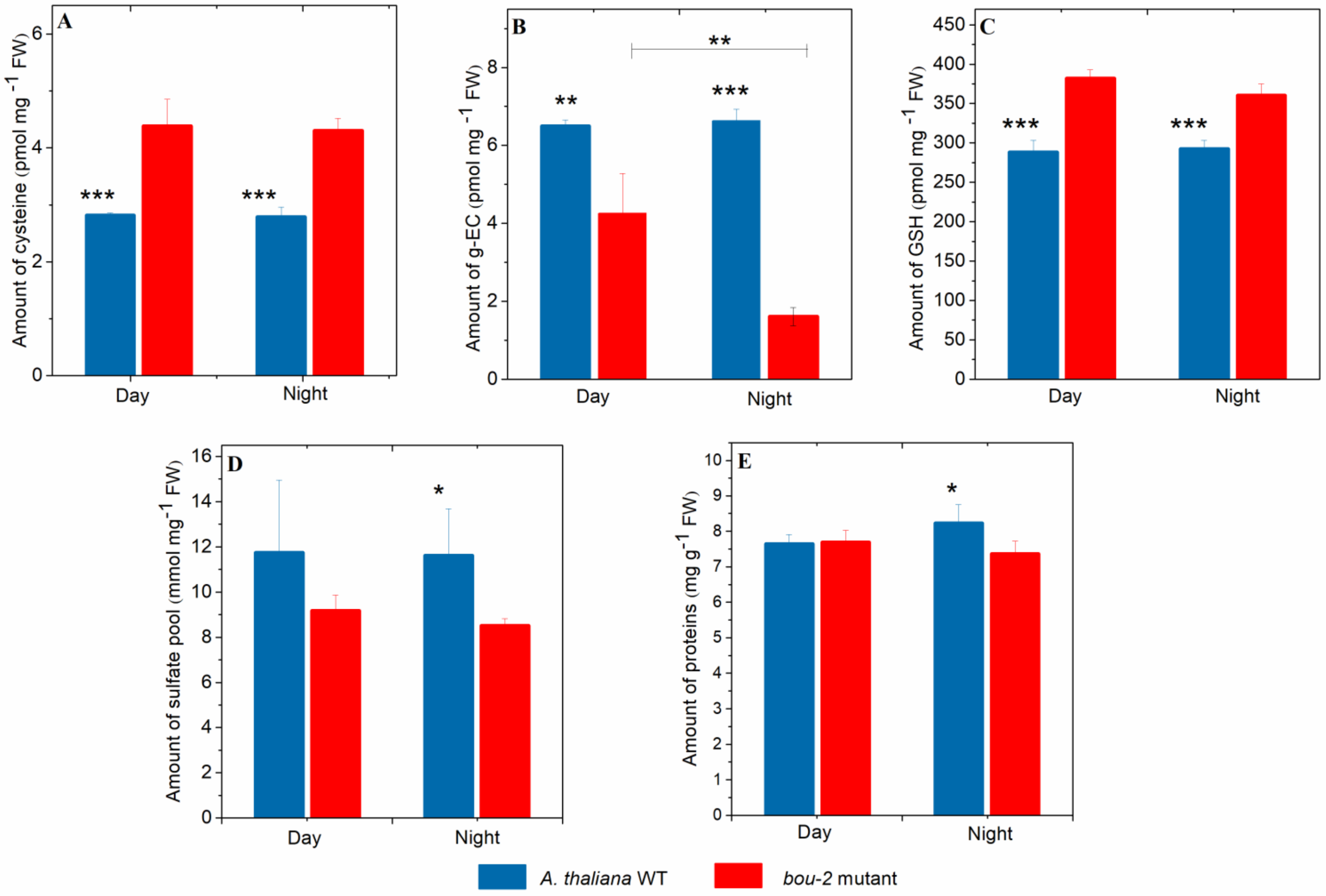

Supplemental Figure S3. Foliar content of Cysteine (A), γ-Glutamincysteine (γ-EC) (B), gluthatione (GSH) (C) sulfate (D) and proteins (E) of *A. thaliana* WT and the *bou–2* mutant during day and night. Plants were grown at elevated (3000 ppm) CO_2 and harvested ca. 10 min after light onset and 3h before light onset. Small letters present significant differences between different plant types within same treatment (P<0.05). Asterisks indicate significant differences between day and night within same plant type (* P<0.05, **P<0.01 and *** P<0.001). All values are means ± standard deviation of 4 replicates.
